# Recovery of Nutrients from Residual Streams Using Ion-Exchange Membranes: Current State, Bottlenecks, Fundamentals and Innovations

**DOI:** 10.3390/membranes12050497

**Published:** 2022-05-04

**Authors:** Natalia Pismenskaya, Kseniia Tsygurina, Victor Nikonenko

**Affiliations:** Membrane Institute, Kuban State University, 149 Stavropolskaya Str., 350040 Krasnodar, Russia; kseniya_alx@mail.ru (K.T.); v_nikonenko@mail.ru (V.N.)

**Keywords:** nutrient, phosphate, ammonium, recovery, membrane-based processing, ion-exchange membrane, fouling, mass-transfer

## Abstract

The review describes the place of membrane methods in solving the problem of the recovery and re-use of biogenic elements (nutrients), primarily trivalent nitrogen N^III^ and pentavalent phosphorus P^V^, to provide the sustainable development of mankind. Methods for the recovery of NH_4_^+^ − NH_3_ and phosphates from natural sources and waste products of humans and animals, as well as industrial streams, are classified. Particular attention is paid to the possibilities of using membrane processes for the transition to a circular economy in the field of nutrients. The possibilities of different methods, already developed or under development, are evaluated, primarily those that use ion-exchange membranes. Electromembrane methods take a special place including capacitive deionization and electrodialysis applied for recovery, separation, concentration, and reagent-free pH shift of solutions. This review is distinguished by the fact that it summarizes not only the successes, but also the “bottlenecks” of ion-exchange membrane-based processes. Modern views on the mechanisms of NH_4_^+^ − NH_3_ and phosphate transport in ion-exchange membranes in the presence and in the absence of an electric field are discussed. The innovations to enhance the performance of electromembrane separation processes for phosphate and ammonium recovery are considered.

## 1. Introduction: Nutrient Sources, Environmental Impact

Nutrients are biologically significant chemical elements necessary for the human or animal organism to ensure normal functioning. Macronutrients are substances whose daily intake exceeds 200 mg. Biogenic macronutrients include hydrogen, carbon, oxygen, sulfur, nitrogen (N^III^) and phosphorus (P^V^), which are necessary for the reproduction of proteins, fats, carbohydrates, enzymes, vitamins, and hormones. Macronutrients, such as potassium, calcium, magnesium, sodium, and chlorine are necessary for building bone tissue or forming the basis of native fluids.

Humanity, which could reach a population of 9 billion [1] by 2037, obtains these nutrients from food derived from animal and vegetable matter. For the cultivation of agricultural crops, mineral fertilizers, which contain nitrogen and phosphorus, are increasingly being used. The most valuable are those that contain N^III^ in the form of ammonium cations, NH_4_^+^, and P^V^ in the form of phosphoric acid anions H_x_PO_4_^(3−x)−^. In 2018, the global market demand for fertilizers amounted to 1.99 × 10^8^ tons and, according to forecasts [2], will further increase by 2% per year. The global fertilizer market in 2020 was over US$171 billion.

It should be noted that the source of P^V^ is mainly sedimentary rocks (primarily fluorapatite, detrital quartz, carbonate cements, etc.), the world geological reserves of which are estimated at about 1.33 × 10^12^ tons [3]. P^V^ resources are distributed very unevenly (74% of the world reserves are in Morocco [4]) and are often located in the northern regions, for example, on the Kola Peninsula (Russia) above the Arctic Circle [5]. According to the data provided by Cordel, et al. [4], 2.1 ± 4 × 10^6^ tons of phosphorus-bearing minerals are mined annually.

Ammonia is traditionally synthesized from nitrogen and hydrogen using catalysts, high pressures, and high temperatures (Haber–Bosch process). Hydrogen is produced by steam reforming of methane or by electrolysis. Nitrogen is extracted from atmospheric air by the cryogenic method [6]. According to [7], more than 160 million tons of ammonia are produced using the Haber–Bosch process per year (about 80% of this amount is used for the production of nitrogen fertilizers). The total energy consumption for the production of a ton of ammonia is about 9500 kWh and increases to 12,000 kWh per ton if H_2_ is generated by electrolysis of water rather than steam reforming of methane [8,9]. In addition, the Haber–Bosch process generates 4–8 tons of CO_2eq_ per ton of N-fertilizer [10]. According to some forecasts [11,12], in the coming years, the energy consumption for the synthesis of ammonia by the Haber-Bosch method may amount to 1–2% of the world’s energy consumption. This large-tonnage extraction of nitrogen from the atmosphere is increasingly affecting the natural nitrogen cycle.

Note that animals and humans assimilate in the form of proteins only 16% of nitrogen from fertilizers. The remaining nitrogen enters the hydrosphere and atmosphere. About 3.4 million tons of phosphorus-bearing minerals enters wastewater annually [4]. Another powerful source of N^III^ and P^V^ emissions into the environment is animal husbandry and poultry. For example, already in 2018, the total number of cattle, pigs, sheep, and goats in Turkey, Spain, France, and Germany was 62, 56, 41 and 40 million heads [13], respectively. According to [14], the content of phosphates in animal and poultry waste ranges from 3.2 (sheep) to 25 (broiler) kg/t, and ammonium from 0.6 (horse) to 6.2 (broiler) kg/t. Pig manure and cattle manure contain about 8 kg/t of phosphates, and 1.2–1.8 kg/t. In addition, manure contains potassium, the concentration of which varies from 3.2 (cattle) to 18 (broiler) kg/t. In addition, phosphates are a constituent of detergents [15], while ammonia and ammonium anions are used in explosives, pharmaceuticals and cleaning agents, and many other industrial processes [16]. Ammonium and phosphates accumulate in the filtrates of municipal solid waste landfills due to natural decay (biochemical decomposition) of the organic phase [17,18,19]. The content of ammonium in the landfill leachates ranges from 2 to 4 kg/t [20].

As a result, phosphates and ammonium enter the environment in abundance from industrial, municipal, and livestock wastewater, and are washed out of agricultural soils. Increasing volumes of industrial, agricultural, and municipal waste due to urbanization do not have time to be processed by bacteria or assimilated by living organisms [21]. As a result, phosphates, ammonium and, to a lesser extent, nitrates accumulate in the hydrosphere, leading to eutrophication and hypoxia of water bodies [22], algal blooms [23], as well as to the development of various pathologies in their inhabitants. For example, an excess of ammonium causes gill disease, convulsions, coma, and death of fish [24]. In addition, ammonia is a greenhouse gas; NH_3_ emissions from aquatic environments contribute to the greenhouse effect [25]. Gaseous decomposition products of nitrogen-containing substances enter into oxidation reactions in the Earth’s ozone layer, which leads to its destruction [26]. About 10–40% of N-fertilizers are converted to N_2_ and partially are transformed into nitrogen oxides, which can affect the process of global warming and atmospheric pollution [6]. Especially dangerous for the environment is N_2_O gas, whose contribution to global warming is 298 times higher than CO_2_ [27].

Thus, a paradoxical situation arose. On the one hand, humanity needs more and more ammonium, phosphates, and other nutrients. Their production consumes nonrenewable resources and/or huge amounts of electricity. On the other hand, these substances in increasing quantities enter the biosphere and cause irreparable damage to it. An elegant solution to these interrelated problems can be the recovery and concentration of ammonium, phosphates and other nutrients from residual streams, and use them for the production of fertilizers [28,29,30]. The development of highly efficient nutrient cycle systems will significantly reduce the anthropogenic and technogenic load on the environment, minimize the shift in the nitrogen cycle of the biosphere, and reduce the fossil phosphorus sources depletion.

## 2. Conventional Methods of the Residual Streams Processing

### 2.1. Classification of Nutrient-Containing Wastes

The recovery and concentration of nutrients from municipal wastewater, landfill leachates, manure, products of biochemical processing of biomass, etc. is an extremely complex multidisciplinary problem. Indeed, the qualitative and quantitative composition of these substances is extremely diverse. Wastes contain solid and liquid phases [31]. In addition, nutrients are often in insoluble forms or associated with heavy metals and other harmful substances [32]. That is why the process of nutrients (in particular N^III^ and P^V^) recovery is multi-stage.

A comprehensive review of nitrogen containing solid residual streams is provided by Deng et al. [6]. They proposed a classification that establishes the relationship between the composition of wastes and the method of their processing.

The first group of the solid residual streams are bio- and food waste, the organic fraction of municipal solid waste, and spent biomass, such as the waste activated sludge from wastewater treatment plants (WWTPs) and algal sludge. The typical total ammonia nitrogen (TAN)—the sum of dissolved ammonium NH_4_^+^ and ammonia NH_3_—content in the solid residual streams is 1 g/kg; the total Kjeldahl nitrogen (TKN)—the sum of organic and TAN nitrogen—are in the range between 3 and 12 g/kg mainly in the protein form [33]. Anaerobic digestion is a widely applied technology to treat these solid residual streams due to relatively high COD (chemical oxygen demand, the oxygen equivalent of the organic matter in a water) >10 g/kg [34].

All types of manure (poultry, cattle, swine, etc.) mainly contain organic nitrogen and P^V^ and form the second group. Their rather high TAN content (1 g/kg for cattle; 2 g/kg for poultry and 4 g/kg for swine manure [35]) hinders aerobic biochemical processing [36]. Therefore, a preliminary TAN extraction is shown for this case.

The third group includes the liquid fraction of raw swine manure (swine liquid), human urine, and landfill leachate. These nutrient sources contain a high portion of total suspended solids TSS ≈ 19 g/L, TKN from 3 to 7 g/L, and TAN/TKN ≈ 0.8.

The fourth group is industrial wastewaters (mining and fertilizer industry, fish/fishmeal processing, glutamate, pectin industries, etc.). Note that the residual streams of the mining and fertilizer industry contain almost no organic impurities. All nitrogen is in the form of TAN with a concentration from 2 to 5 g/L [6]. In other cases, the mixtures from which nitrogen must be removed are more complex. At the same time, their composition often turns out to be less diverse than for the first three groups of wastes.

Deng, Z., et al. [6] divide all the residual streams into three categories: TAN/TKN < 0.5, TSS and COD > 24–36 g/kg (category 1); TAN/TKN ≥ 0.5, TSS > 1 g/L (category 2); TAN/TKN ≥ 0.5, TSS < 1 g/L (category 3). Category 1 requires the mandatory transformation of organic nitrogen and phosphorus into inorganic N^III^ and P^V^ while reducing TSS and COD. Category 2 must be refined of TSS before TAN recovery. Category 3 allows the recovery of nutrients without preliminary separation of TSS.

**Figure 1:**
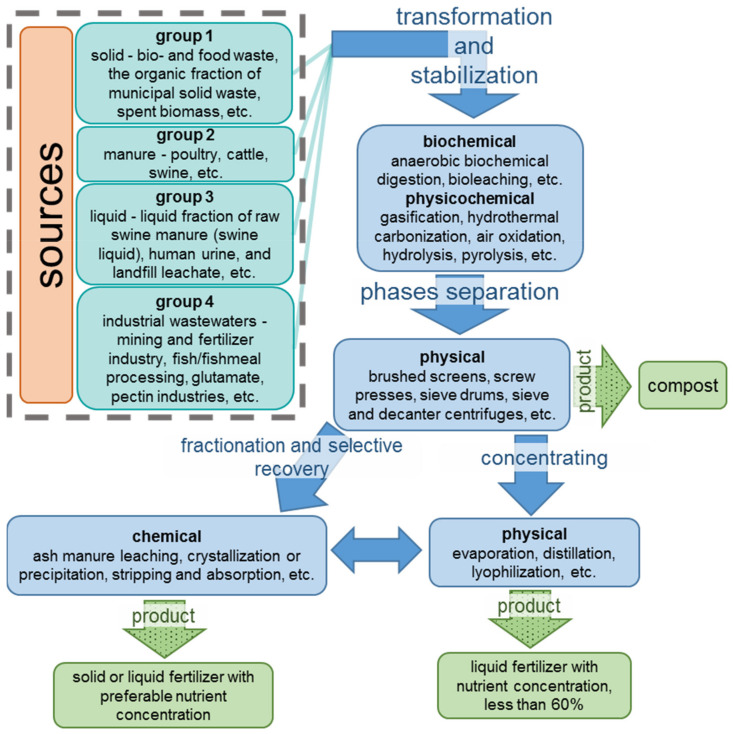
An overview of residual streams and their processing steps.

A scheme presented in Figure 1 contains the main sources of nutrients and the residual streams processing stages.

### 2.2. Stabilization of Wastewater and Transformation of Nutrients

The first stage is designed to stabilize wastes and convert nutrients into forms suitable for their further processing. A detailed description of the processes used at this stage can be found in reviews [6,14,31,37,38]. We will only mention a few of these processes.

*Biochemical methods*, in particular anaerobic biochemical digestion (AnD), are the most common. The result of anaerobic microorganism activity in an anaerobic reactor is the conversion of organic substances into methane, carbon dioxide, hydrogen sulfide, ammonium, and other volatile compounds [39,40]. Livestock manure AnD is attractive due to energy recovery from biogas production, as well as pathogen reduction and hydrolysis of organic solids [41]. Bioleaching is based on the ability of some microorganisms to grow in acidic conditions and perform oxidation with the release of heavy metals and nutrients solubilization from solid substrates [42]. In addition to the gas phase, solid digestate and reject water (liquid fraction of the digestate) are products of biochemical processing, in which the TAN/TKN ratio reaches 0.9. Organic phosphorus is partially converted into a soluble inorganic form [43]. Moreover, the electrochemical treatment of waste activated sludge before the process of its anaerobic fermentation provides an increase in the content of organic and inorganic phosphorus in the liquid phase [44].

The methods (co-digestion, pre- or side treatment, addition of methanogenic culture, side-stripping removing of NH_3_ using high temperature and/or pH more than 8, etc.) that can increase the effectiveness of AnD are described in review [6]. According to calculations made by Kevin et al. [45] the nutrient loadings (ton/day) to anaerobic digesters in the 2020 year were 117 (TAN) and 76 (total P^V^). In 2050, these parameters will increase to 195 (TAN) and 122 (P^V^). Biochemical methods are relatively inexpensive [46], but require a long residence time (several weeks) due to the slow kinetic of the biochemical process. In addition, bioreactors occupy large areas and cause greenhouse gas emissions. The content of N_2_O in this gas can reach 80% [47].

*Physicochemical processes* (gasification, hydrothermal carbonization, air oxidation, hydrolysis, pyrolysis, etc.) allow converting biomass into gases and ash residues [38]. The use of some of these methods (for example, incineration [48]) causes the ash to be enriched with phosphorus while nitrogen enters the gas phase. Ash may contain from 11 to 23 wt. % P_2_O_5_ and about 2 wt.% potassium, which is comparable to their content in phosphate rocks [49]. The use of these methods to transform nutrients into a form convenient for further processing requires significantly less time. For example, the air oxidation method requires from several seconds to several minutes and provides up to 80–90% conversion of organic nitrogen to TAN [46]. However, large energy and chemical inputs, as well as more complex reactor designs, are needed.

### 2.3. Phases Separation

The second stage consists in the separation of the gas, liquid, and solid phases. Biogas is collected, purified and then used for energy production [50]. Brushed screens, screw presses, sieve drums, and sieve and decanter centrifuges are used in separation processes [51]. Sancho et al. [52] suggest using direct filtration to recover nutrient-containing organics from various streams. Some non-mechanical methods, such as the addition of flocculants, can improve separation efficiency [53].

### 2.4. Nutrient Concentration

**Figure 2:**
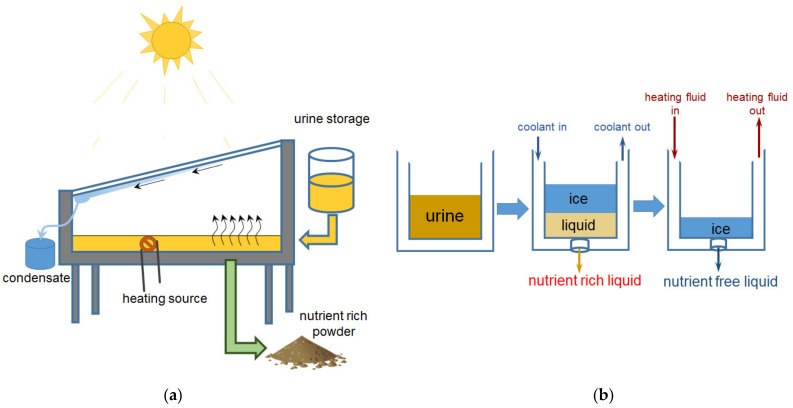
Concentration of liquids containing nutrients by evaporation (**a**) and lyophilization (freezing) (**b**). Based on [56].

The third stage includes the concentration of nutrients. The simplest method is evaporation (Figure 2a), which, for example, allows one to extract 95% of the water from urine [54] by heating using coil or solar energy.

*Lyophilization/freeze concentration (FC)* separates water from liquid by ice crystallization at low temperature, followed by ice removal from the concentrate [55] (Figure 2b).

Lowering the temperature leads to enrichment of the solution with nutrients and demineralization of ice due to the difference in vapor pressure in salt and pure water. A review of these methods is given in [14]. Thus, Cantero et al. report [57] that the use of FC processes makes it possible to extract up to 50% of water from manure. The freezing-thawing process concentrates up to 60% of the nutrients that were in the manure [58]. Up to 99% of the nitrogen in the urine could be recovered at a temperature of −30 °C. However, achieving such a low temperature requires additional energy consumption [59]. Dadrasnia et al. [14] believe that the use of FCs could be a useful addition to hybrid nutrient recovery technologies.

### 2.5. Fractionation and Selective Recovery of Nutrients

The fourth stage aims fractionation and recovery of nutrients. Traditionally, chemicals and/or high energy costs are required for its implementation.

*Chemical methods*. An example of the application of chemical methods is the using dilute hydrochloric or sulfuric acids to extract phosphorus and potassium from the manure ash [60,61]. The use of sulfuric acid is preferred because the resulting solution is enriched in phosphoric acid and contains a small amount of calcium due to precipitation of CaSO_4_ [62]. An increase in the acid concentration promotes an increase in concentration of phosphorus (in solution) extracted from the ash [61].

*Crystallization/precipitation-based technologies* include struvite (MgNH_4_PO_4_∙6H_2_O) complex fertilizer precipitation, which is one of the most common and studied methods for extracting ammonium and phosphates from pre-concentrated liquid digestate. The method is based on the addition of magnesium chloride, or sodium hydrogen phosphate, or alkali to the digestate sludge supernatant. There are many patents and scientific papers devoted to improving the performance of this process. Reviews are made by Shi et al. [38], Li et al. [63], Larsen et al. [64], Yakovleva et al. [65], and Krishnamoorthy et al. [66]. The method is quite simple and allows one to obtain fertilizers from residual streams of various composition. Examples of the commercialization of this method at industrial and municipal wastewater treatment plants are presented in Ref. [67].

The disadvantages of the method are: secondary emissions into the environment caused by the introduction of chemicals to ensure precipitation and the necessary values of pH 8.0–9.5 [68]; additional costs for acquiring chemicals, as well as for their safe transportation and storage; large areas occupied by chemical reactors. In addition, the preliminary concentration of phosphates to 100 mg/L and more [69] with an average content in untreated secondary streams from 8 mg/L to 60 mg/L [63] is needed.

An alternative P^V^ precipitation method is to obtain slow-release fertilizer vivianite (Fe_3_(PO_4_)_2_ 8H_2_O, which can be used to produce LiFePO_4_ used in Li-ion batteries [70]. According to Ref. [68], vivianite has a more attractive market price (of the order 10 thousand euros per ton) compared to struvite (from 100 to 500 euros per ton). However, obtaining chemically pure vivianite requires magnetic separation, centrifugation, extraction of organic matters, etc., which significantly increase the cost of the process. In the case of processing industrial wastewater that contains practically no NH_4_^+^ − NH_3_ (for example, in phosphoric acid production or in anodizing industry), P^V^ precipitates as hydroxyapatite (Ca_5_(PO_4_)_3_(OH) or similar substances [71]), whose value in agriculture and industry is less high.

Note, struvite precipitation makes it possible to extract 75% or more of phosphates but is much less effective in relation to ammonium. The fact is that N^III^ partially (6 < pH < 12) or completely (12 < pH) is in the form of volatile NH_3_ [72].

The *thermal distillation* method is more attractive for recovery of volatile components from liquid substances [73]. This process can be carried out continuously. The disadvantages of the method are the complexity and bulkiness of distillation columns design and high energy costs for heating. According to estimates presented in [64], the energy demand of distillation is around 110 Wh/L.

*The ammonia stripping and absorption method* involves heating a liquid with a pH of 8–12 to a temperature of 60–80 °C [74,75]. In this case, NH_4_^+^ in the fluid is transformed into NH_3_ and volatilizes from it into the air flow. An ammonia-containing gas stream is bubbled through nitric, sulfuric, or phosphoric acids to produce liquid fertilizers (ammonium sulphate, phosphate, or nitrate). Examples of full-scale commercialization of this process are reported in Ref. [76]. The production of such biofertilizers is environmentally attractive, especially if aggressive acids are replaced with the most sustainable (citric acid, for example [77]). Vaneeckhaute et al. [75] note that the ammonia stripping and absorption process requires less capital expenditure than ammonium recovery using other methods. However, the benefits of this process depend largely on the method of pH increasing in the treated liquid.

## 3. Modern Trends in Nutrients Recovery

### 3.1. The Place of Membrane Processes in the Circular Economy of Nutrients

**Figure 3:**
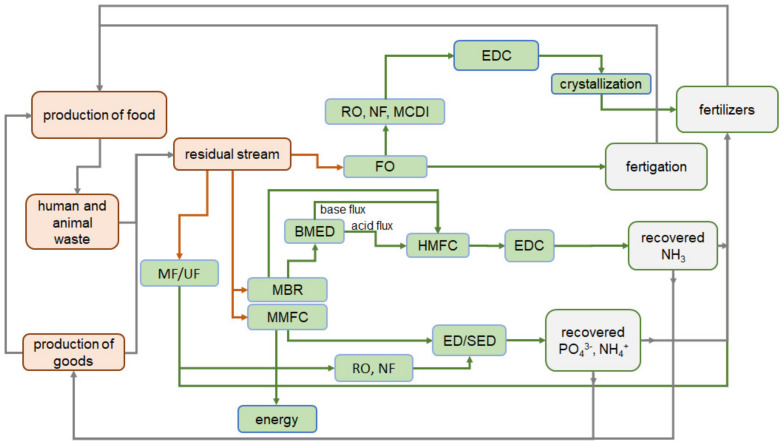
A scheme of some possible steps for nutrient recovery and recycling using membrane technologies.

Modern trends in the involvement of N^III^ and P^V^ in the Circular Economy are comprehensively described in reviews [78,79,80]. They are mainly focused at replacing traditional methods of nutrient recovery with membrane methods and at developing multi-stage hybrid processes using membranes. An analysis of the reviews of recent years leads to the conclusion that almost all membrane technologies are used to solve this problem. External pressure-driven, electric field-driven, vapor pressure-driven, chemical potential-driven membrane technologies are among them [81]. Until recently, the commercial application of membrane technologies was fragmented and limited by the high cost of membranes [33]. At the same time, the increase in the production of membranes in recent years gives hope for a decrease in their cost. In this case, membrane technologies will become economically competitive compared to traditional technologies. A scheme (Figure 3) contains some possible steps for nutrient recovery and recycling using membrane technologies.

Note that the currently developed membrane processes are quite difficult to classify by stages, in contrast to traditional methods (Section 2). This is due to the multifunctionality of membrane modules, each of which, as a rule, simultaneously performs several functions. Transformation of nutrients and their separation; generation of bioelectric energy and selective recovery of individual components; neutralization of liquid effluents; and concentration of nutrients are often combined.

### 3.2. Main Types of Membranes

*Microfiltration (MF) and ultrafiltration (UF)* porous membranes may be made of inorganic (porous titanium, aluminum, and zirconium oxide, etc.) and organic polymeric (fluoroplastic, cellulose esters, polyamide etc.) materials. They have an effective pore diameter 0.5–20 µm (MF) and 0.01–0.1 µm (UF). A low-cost sheet of carbon felt [82] can perform functions similar to MF and UF in membrane bioreactors (MBR) and membrane microbiological fuel cells (MMFC). These membranes are used to retain solid particles and liquid droplets, colloidal species, and bacteria, as well as separation from solutions of viruses and macromolecular substances with a molecular weight of the order of several thousand. The separation is mainly done by the sieving mechanism. Micro- and ultrafiltration is carried out at relatively small operating pressure differences: 0.01–0.2 MPa (MF) and 0.1–0.5 MPa (UF) [63].

*Nanofiltration (NF) and reverse osmosis (RO)* membranes are mainly made of hydrophilic and hydrophobic polymeric materials. Moreover, the selective layer deposited on a large porous substrate has pores with an effective diameter of 0.5–10 nm (NF) and about 1 nm (RO). Polar carboxyl, sulfone, or amino groups are located on the pore walls of NF and RO membranes, providing Donnan exclusion of coions, which have the same electrical charge as the fixed groups. The electrostatic mechanism (NF) and the formation of an electric space charge between the inlet and outlet of pores (RO) [83] are the main mechanisms for the retention of macromolecular substances with molecular weights from several hundred to several thousand Daltons (NF) and organic substances with a molecular weight of less than a few hundred Daltons (RO). In addition, these mechanisms are implicated in separation of multiply charged organic and inorganic ions from smaller neutral or singly charged species. The operating pressure differences is 0.5–1.5 MPa (NF) and 1–10 MPa (RO) [63]. In all baromembrane processes, the electrostatic and adsorption mechanisms increase their contribution to the separation of substances as the pore sizes decrease.

*Forward osmosis (FO) membranes.* The structure of inorganic and organic osmotic membranes is similar to RO membranes. The difference lies in the obligatory hydrophilicity of the selective layer. For example, membranes can be made of cellulose triacetate with an embedded polyester screen [84].

*Gas separation membranes (GSM), including hollow fiber membranes (HFM)* consist of a porous polymer that has a complex asymmetric structure. A polymer density increases as it approaches the outer gas separation layer. The membranes are made of hydrophobic synthetic materials (for example, polytetrafluoroethylene (PTFE), polyvinylidene fluoride (PVDF), or polypropylene (PP)). [85]. The high surface tension of water prevents the liquid phase from entering the pores of the hydrophobic polymer.

*Ion-exchange membranes (IEMs)* can be made of hydrophilic and hydrophobic homogeneous or composite [86] materials and have pores from a few nanometers to several micrometers (see reviews [87,88]). Their main difference from other membranes is the high concentration of polar groups. These fixed groups cover the membrane surface and the pore walls uniformly distributed over the membrane bulk. Cation-exchange membranes (CEM) contain negatively charged sulfonate or phosphonate, or carboxyl fixed groups and selectively transfer cations. Anion-exchange membranes (AEM) typically contain positively charged quaternary ammonium bases or weakly basic secondary and tertiary amines. They selectively transport anions under the action of concentration difference and/or electrical potential drop. The selectivity of CEM and AEM is mainly determined by the Donnan exclusion of coions from the diffuse part of the electric double layer formed on the pore walls by fixed groups and counterions (ions with an electrical charge opposite to the charge of fixed groups). Bipolar membranes (BPMs) consist of cation and anion-exchange layers and are intended for reagentless generation of H^+^, OH^−^ ions due to water splitting (WS) at the CEM/AEM interface.

### 3.3. Membrane Bioreactors and Membrane Microbiological Fuel Cells

**Figure 4:**
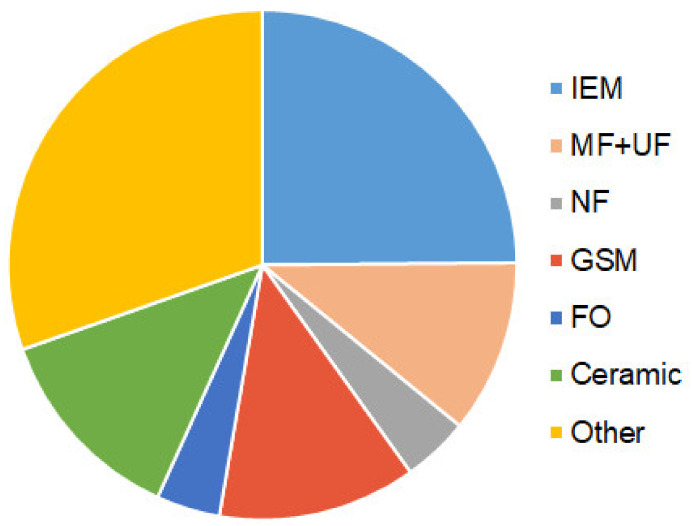
The share of publications in Scopus (reference dated 3 April 2022) devoted to the development of MBR and MFC equipped with ion-exchange (IEM), micro- (MF) and ultrafiltration (UF), nanofiltration (NF), gas-separation (GSM), osmotic (FO), and other membranes, including ceramic membranes.

Just as in the case of traditional methods, biochemical methods are mainly used to stabilize wastewater and convert nutrients into forms convenient for their further processing. Meanwhile, the use of osmotic [89,90], microfiltration and ultrafiltration polymeric and ceramic membranes [91,92,93], as well as reverse osmosis [94], nanofiltration [95,96], ion-exchange [86], or gas separation [97] membranes in MBRs and MMFCs allows selective and reagent-free recovery of target components even if their concentrations in liquid or gaseous phases are low. MMFCs combine two processes: the transformation of nutrients from complex organic substances into simple inorganic forms and the generation of electricity through the simultaneous implementation of redox reactions involving microorganisms. Interest in the development of these methods is extremely high. Indeed, a search in Scopus for the keywords “membrane bioreactor OR membrane fuel cell” yields 17,991 publications (reference dated 3 April 2022). Moreover, only in 2021, 4910 articles were published. The largest number of biochemical devices described in publications contains IEM (17990 pcs.), MF and UF (7960 pcs.), NF (3070 pcs.), FO (2920 pcs.), as well as flat and hollow fiber gas-separation membranes (9030 pcs.) (Figure 4).

*Anaerobic Membrane Bioreactors (AnMBRs)* do not require oxygen for transforming biodegradable organic substances as compared to aerobic bioreactors and produce less solid waste that requires further processing. These circumstances, as well as the generation of combustible gases that are used as biofuels, make AnMBR cheaper and more attractive in comparison to aerobic biochemical methods [98]. Anaerobic digestion mineralizes organic phosphorous and nitrogen in the form of H_x_PO_4_^(3−x)−^ and NH_4_^+^ − NH_3_, which accumulate in digestates, can be used in fertigation (the application of liquid complex fertilizers, simultaneously with the irrigation of agricultural land). Such use can significantly reduce the environmental impact of AnMBRs related to eutrophication of natural water bodies [99]. However, it should be noted that the concentration of nutrients in the solid and liquid phases of the digestate is not too high. Therefore, a deeper processing of the digitate sewage sludge and nutrient-containing wastewater looks more promising.

Porous (MF and UF) membranes in AnMBR make it possible to separate the liquid and solid phases according to the sieving mechanism, i.e., retaining in the reactor species (including viruses, antibiotic-resistant bacteria and the pathogens [100,101]), whose sizes exceed the sizes of the membrane pores. The use of FO membranes [102,103] contributes to the removal of water and the accumulation of phosphates, ammonium and hardness ions in the bioreactor. The use of Ca^2+^ and/or Mg^2+^ containing draw solutions allows the precipitation of concentrated nutrients without addition of extra chemicals. Such osmotic MBR is characterized by lower energy consumption, less severe membrane fouling, and high retention of soluble nutrients in suspended liquor compared to other bioreactors [104]. IEMs are used for the selective recovery of phosphoric acid anions and ammonium cations from MBR liquid digestate [105,106].

Robles et al. [98] believe that AnMBR in combination with fertigation is a membrane technology that can already be “implemented for full scale low-loaded water treatment”. However, for its implementation, it is required to study some aspects of water reuse [107].

**Figure 5:**
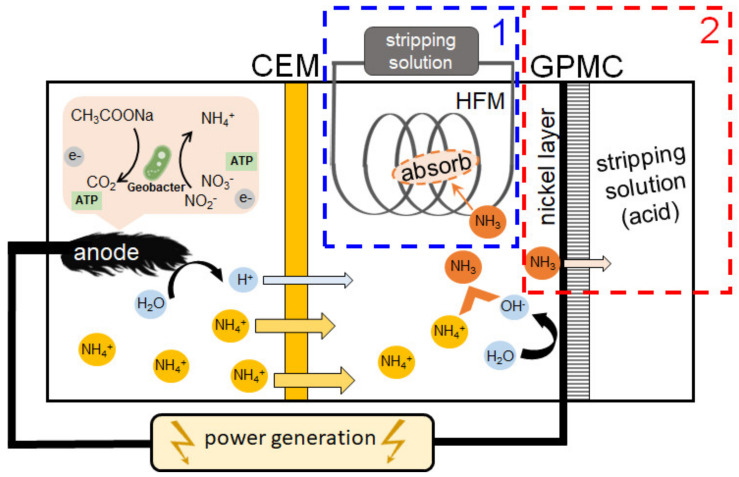
Schematic diagram of an MMFC for energy production and NH_3_ volatilization, which contains cation-exchange membrane (CEM) and hollow fiber gas separation membrane (HFM) (1) or flat gas-permeable membrane cathode (GPMC) (2). Adapted and modified from [108,110].

*Microbiological fuel cells (MFCs)*, which are reviewed in [64,80], use special types of bacteria that are able to oxidize organic substances with the release of electrons and the conversion of nitrates and nitrites into ammonium cations [108] (Figure 5). The use of CEM makes it possible to ensure the selective transport of these cations to the cathode, on the surface of which OH^−^ ions are generated due to electrochemical WS. The alkaline environment promotes the deprotonation of ammonium cations with the formation of volatile ammonia: NH_4_^+^ + OH^−^ = NH_3_∙H_2_O. The latter can be obtained from the cathode solution using hollow fiber gas separation membranes (hollow fiber membrane contactor (HFMC)) immersed in the cathode compartment [109] or through gas-permeable membrane cathode (GPMC) having the surface coated with a hydrophilic nickel-containing layer [110] (Figure 5).

The use of a gas-permeable membrane cathode makes it possible to increase current density and reduce energy consumption by 11% and 20%, respectively. At the same time, the NH_3_ recovery rate increases by 40% as compared to the conventional cathode configuration. Ammonia that has penetrated into the bulk of the gas-liquid contactor or transferred through a gas-permeable cathode is fed into a separate container and absorbed by a sulfuric (or other) acid solution to form (NH_4_)_2_SO_4_ [109,110,111].

As shown by Xue et al. [90], the integration of FO into MFC allows a 19% increase in power density in osmotic microbiological cells (OMFC) due to the water-flux-facilitated proton transfer. The application of a magnetic field promotes the formation of a biofilm on the MFC anode and can increase the current density by 20–30% in the case of OMFC [112].

Currently, a great number of articles describe the successful use of mixed bacterial communities that form biofilms on the cathode and anode. The addition of these microalgae communities, which are in the form of a suspension in the cathode compartment and as a biofilm on the cathode [82,92,93], contributes to an increase in the current density achieved and a more complete denitrification of the waste.

MMFC are widely investigated to recover N^III^ from wastewater [113], landfill leachate [82] and urine [114,115,116], where the conversion of urine to ammonium via ureolysis is accelerated by a generated electric field. The presence of CEM and AEM in such cells makes it possible to separate P^V^ (which is in the form of phosphoric acid anions) and NH_4_^+^ cations. Preliminary partial recovery of P^V^ from urine contributes to an increase in the current density generated by MMFC [112]. An example of the use of energy generated in a microbial fuel cell to recover P^V^ from sewage sludge digestate is presented in Ref. [117]. This energy is required to dissolve the precipitated iron phosphate, and then convert the phosphate anions to struvite.

The use of microbial electrolysis desalination with electrochemically active bacteria in the anode compartment, CEM and AEM allows achieving the feed solution desalination by 73% and recover up to 83% N^III^ in the form of NH_3_·H_2_O. The generated bioenergy compensates up to 58% of the energy costs for this process [118].

**Figure 6:**
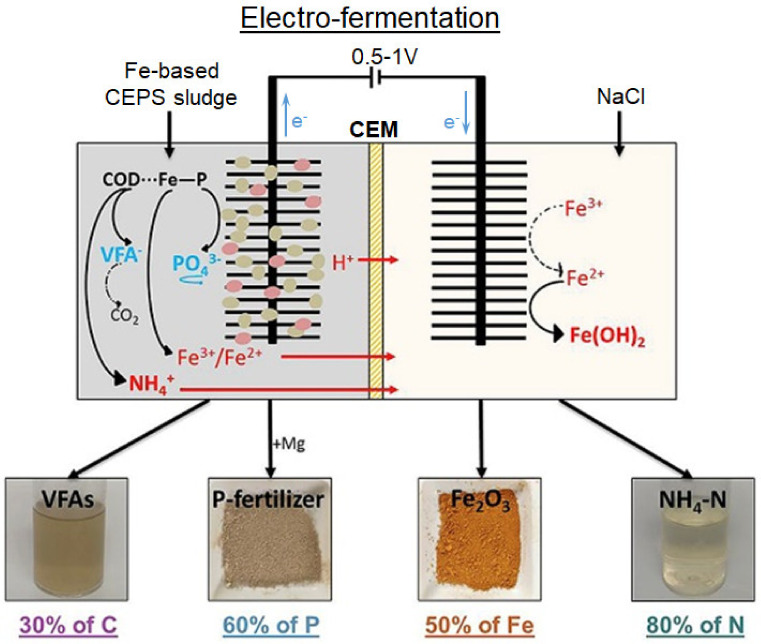
Electrofermentation of sludge that contains P^V^ and iron in organic matter. Reproduced with permission from [119]. Copyright 2022 Elsevier.

*Electrofermentation* of sludge that contains P^V^ and iron in organic matter (COD∙∙∙P∙∙Fe) was carried out by Lin et al. [119] using an electrolyzer whose anode and cathode compartments were separated by a cation-exchange membrane (Figure 6). A community of microorganisms (Firmicutes, Bacteroidetes, Proteobacteria), located on the anode, transforms insoluble organic substances into soluble inorganic forms PO_4_^3−^ and NH_4_^+^, Fe^3+^/Fe^2+^. The effect of an electric field (0.5–1.5 V) increases the activity of microorganisms. As a result, P^V^ dissolution increases from 8% to 56%. The NH_4_^+^, Fe^3+^/Fe^2+^ cations as well as the protons generated at the anode are transferred through the CEM to the cathode compartment. Phosphate-enriched supernatant may be used as fertilizer.

MBR and MFC studies are still carried out mainly on laboratory samples. However, the successes of recent years (pilot-scale demonstrations of microbial electrochemical technologies), described in the review [120], allow one to hope for a transition to industrial-scale facilities in the coming years. The cheaper membranes and electrodes, as well as the gaining of new knowledge about the microbe-electrode interaction, will facilitate the acceleration of this transition.

Membrane processes for nutrient recovery from liquid fractions obtained after biochemical processing steps are very diverse. We will discuss them in Section 3.4, Section 3.5, Section 3.6, Section 3.7.

### 3.4. Recovery of Volatile Fractions (NH_3_) Using Gas Separation Membranes

*Membrane distillation (MD)* is a thermally driven separation process in which separation occurs due to a phase change. The hydrophobic membrane acts as a barrier to the liquid phase, allowing the volatile phase (e.g., NH_3_, water vapor, volatile organic compounds) to pass through the membrane pores [121]. The driving force of the process is the partial vapor pressure difference, usually caused by the temperature difference. In the case of spiral wound MD module, the energy costs are from 180 to 240 Wh/L for treating water with salinity about 35 g/kg [122]. If vapor-compression distillation and heat recovery are applied to partially nitrated urine, the energy consumption is 10^7^ Wh/L [123]. The advantage of MD is the ability to use low-grade heat, while pre-vapor-compression distillation uses electricity. In recent years, MBR combined with MD unit has been increasingly used. An overview of such studies can be found in [97].

The use of gas-permeable membranes in the *vacuum membrane stripping process (VMS)* allows to reduce the installation volume due to the large gas/liquid exchange area [124] and to achieve 1–11% NH_3_ concentration in the gaseous NH_3_-H_2_O mixture. This concentration of ammonia is sufficient for use in solid oxide fuel cells SOFC) to generate electricity without emission of oxidized nitrogen oxides into the environment. The electricity generated in the fuel cell (9 MJ/kg N) is enough to cover its demands (7 MJ/kg N) for NH_3_ recovery from residual waters. Rivera et al. [125] report that the use of hydrophobic (polytetrafluoroethylene) flat sheet membranes with a pore radius of 220 nm leads to the 71.6% extraction of ammonia in the H_2_SO_4_ stripping solution at 35 °C.

Liquid-liquid HFMC, which is called for short *liquid-liquid membrane contactor* (LLMC) is a device that implements the separation process between a gas-containing liquid and an adsorbent liquid (or chemisorbent). The use of LLMC [64,126] makes it possible to recover up to 98–99% of ammonia from urban wastewater and liquid digestate as a post-treatment for an anaerobic bioreactors. The driving force of the process is the chemical potential difference on both sides of the membrane. The hydrophobic (PP or PTFE) membranes in LLMC are a barrier to inorganic and organic micropollutants. At the same time, NH_3_, which is a gas, is transferred through their pores, from the feed solution to the acid (HNO_3_, H_2_SO_4_, H_3_PO_4_) stripping solutions, and participates in the formation of ammonium salts of these acids. Ammonia transport is driven by the pressure (concentration) difference in ammonia vapor between membrane sides facing the feed and acid stripping solutions. However, a similar pressure (concentration) difference also occurs for water vapor. The associated transport of both ammonia and water vapor limits the concentration of ammonium in the stripping solution [127], which, as a rule, does not exceed 5–8 wt%, while the ammonium concentration of 15–32 wt% is commercially attractive to use in agriculture for fertigation [128]. Therefore, it is reasonable to use LLMC in combination with electrodialysis (ED) concentration process [126] (Section 3.7).

### 3.5. Forward Osmosis and Baromembrane Processes

*Forward Osmosis* is typically implemented to concentrate nutrients in waste streams or digestates [129,130,131]. FO membrane separates the feed solution (wastewater or digestate) and the more concentrated draw solution, which contains non-toxic, low molecular weight substances (sucrose, inorganic salts with precipitation-forming ions, etc.). Due to the difference in chemical potentials, water moves into the draw solution from the feed solution, and the substances dissolved in the draw solution move in the opposite direction. The pressure difference in the FO process is not superimposed. Reviews [63,132] summarize articles devoted to various aspects of FO use in the N^III^ and P^V^ recovery. In particular, the use of Mg^2+^ or Ca^2+^ salts [133] as well as sea water [134] in draw solutions seems to be very promising. Getting into the feed solutions, these cations contribute to the precipitation of struvite or calcium phosphate [63,132]. Almoalimi et al. [135] reported that the flux of ammonium cations in the draw solution decreases if this solution contains highly hydrated divalent cations (for example, Mg^2+^). Non-ionic substances (glucose, glycine, and ethanol) minimize the ion-exchange in the FO membrane. As a result, the rejection of ammonium to feed solution achieves 98.5–100%. There is no pressure drop or potential drop, so the method does not require complex equipment and high energy consumption; FO membranes are less prone to fouling compared to membranes for other applications.

*Nanofiltration and reverse osmosis* can be used to separate multiply charged phosphates and singly charged nitrates or ammonium cations in the processing of FO draw solutions [135,136], anaerobic digestates [137], human urine [138,139], or urban wastewater [140]. Mechanisms based on the Donnan exclusion of coions provide rejection of large, highly hydrated, multiply charged phosphate anions and their concentration in the retentate, while smaller singly charged anions (e.g., NO_3_^−^, NH_4_^+^) are transferred to the permeate. Shutte et al. [141] showed that an increase in the pH of the processed liquid causes an increase in the electric charge of phosphoric acid anions, contributing to an increase in the P^V^ retention rate. The simultaneous application of a pressure drop and an electric field (using electronanofiltration) intensifies the process of recovering ammonium from the galvanic solution, including by alkalizing the solution from the side of the cathode compartment [142].

In the literature, one can find evidence of the successful application of nutrient recovery and concentration using RO [143], UF-RO [144], MF-NF [145], or NF-RO [20] processes. For example, Samantha et al. [145] reported that the MF-NF treatment train in a dead-end filtration system produced a particle-free product water from raw pig manure. Moreover, MF blocks up to 98% TSS, and NF or MF permeate provided 50–70% K^+^ and ammonium retention. Grossi et al. [144] showed that the UF-RO pilot-scale treatment of a gold mining effluent from the blasting stage can recover up to 80% nitrogen compounds at 6 bar. However, according to Gui et al. [146] NH_4_Cl recovery by RO using a single module requires an operating pressure above 30 bar if the solution contains 5 g/L NH_4_Cl, and the concentration of ammonium must be reduced to the discharge standards. Therefore, a series of multiple NF and RO modules are needed to concentrate nutrients to commercially viable concentrations [20], which adds complexity of process control.

### 3.6. Electrochemically Induced Precipitation (NH_4_^+^, PO_4_^3−^) and NH_4_^+^ Transformation to N_2_ or Nitrates

Comprehensive reviews of research aimed at electrochemically induced NH_4_^+^ and PO_4_^3−^ precipitation and NH_4_^+^ transformation to N_2_ or nitrates are given in recent papers [80,147,148,149]. For example, research aimed at processing non-ortho P compounds seems promising. The combination of anodic or anode-mediated oxidation makes it possible to transform this phosphorus into phosphates and precipitate it in cathode compartments [147]. Using of soluble magnesium anodes and the selective transport of the resulting Mg^2+^ cations through the CEM to the cathode compartment allows the precipitation of MgKPO_4_ (which is a buffered fertilizer) while removing NH_4_^+^ [150]. In recent years, electrochemically driven struvite precipitation using a sacrificial Mg anode has been actively developed. Bagastio et al. [148] comprehensively analyze the effect of solution pH, applied current and material composition on magnesium dissolution rate and phosphate removal efficiency. Therefore, we will not dwell on these problems in detail.

### 3.7. Capacitive Deionization and Electrodialysis

#### 3.7.1. Nutrients Recovery and Concentration

**Figure 7:**
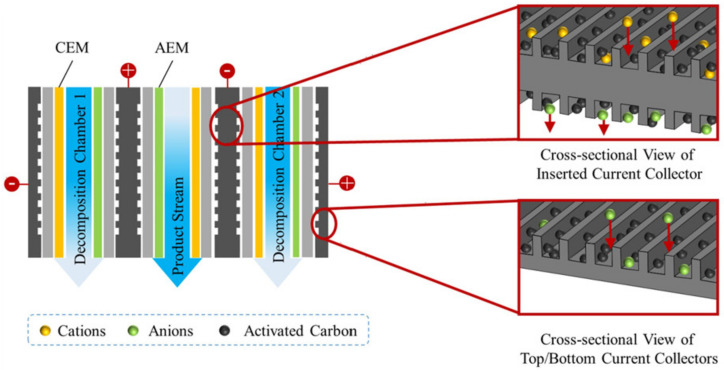
Scheme of the MFCDI system stack design with CEM and AEM membranes. Reproduced with permission from [153]. Copyright 2022 Elsevier.

The essence of a *membrane capacitive deionization (MCDI)* is the adsorption of cations on the cathode surface and anions on the anode surface in an applied electric field (stage 1) and desorption of these ions after this field is turned off (stage 2). This method allows recovering ionic impurities from multicomponent solutions (stage 1) and concentrating them at stage 2. IEMs selectively transfer cations towards the cathode and anions towards the anode (Figure 7) increasing the current efficiency [151,152,153,154]. MCDI has been applied not only on synthetic wastewater, but also on actual municipal wastewater, and has demonstrated a fairly high removal efficiency equal to 39% (NH_4_^+^), 47% (Mg^2+^), and 33% (Ca^2+^), significant desorption efficiency (≈90%) and low energy consumption (1.16 kWh/m^3^) [151]. The increasing of the surface area and the use of flow electrodes (MFCDI) significantly enhance the efficiency of the ion adsorption-desorption processes [152,153].

This method is characterized by relatively low energy consumptions. However, selective electrode coating or selective ion-exchange membranes are required to increase the current efficiency of the target components, N^III^ and P^V^. Some of the examples of using MCDI to nutrients recovery are presented in Section 3.7.3 and Table 1.

*Electrodialysis apparatuses* contain a stack of ion-exchange membranes between the cathode and the anode. Reviews on the application of this method for recovering and concentrating nutrients can be found in Refs. [56,78,80,81,113,155]. Table 1 summarizes some recent research in this field.

**Table 1:** Examples of some membrane systems used for nutrient recovery.

Method	Experiment Details	Feed Solution	Results Achieved	Bottlenecks	The Objective	Ref.
MFCDI	Three-chamber reactor consisting of cathode, anode, two AEM (TWEDA-I), and CEM (TWEDC-I) membranes (TIANWEI, China) separated by a nylon sheet. The flow-electrode: graphite carbon 5 wt%. Membrane surface, S = 48.6 cm^2^, Current density, I = 10 A m^−2^ (charging stage), t = 120 min (charging stage t = 30 min (discharging stage), t_tot_ = 7.5 h	Synthetic urine: prepared with ∼1200 mg L^−1^ NaCl and ∼720 mg L^−1^ Na_2_HPO_4_·12H_2_O	Recovery efficiency per cycle: 164 mg L^−1^ P^V^. Selective recovery factor for P^V^ versus Cl^−^: 2. Energy consumption: 27.8 kWh kgP^V^	Migration uncharged H_3_PO_4_ from anode chamber	Selective recovery of P^V^	[156]
MFCDI	Three-chamber reactor consisting of cathode, anode, CEM (CMI), and AEM (AMI) membranes (Membrane International INC, Ringwood, USA) with nylon spacer between them. The flow-electrode: activated carbon powder (particle size ∼10 μm, Yihuan Carbon Inc.) mixed in 3.55 g L^−1^ Na_2_SO_4_ solution. S = 11.7 cm^2^, Voltage, U = 1.2 V (charging stage), t_tot_ = 7 h	Synthetic wastewater: 40 mg L^−1^ (NH_4_Cl), 30 mg L^−1^ (NaH_2_PO_4_·H_2_O), 30 mg L^−1^ (Na_2_HPO_4_·7H_2_O), 120 mg L^−1^ (NaNO_3_), 200 mg L^−1^ (Na_2_SO_4_)	Removal efficiency: 70−98.5% (salinity), 49−91% (PO_4_^3−^), 89−99% (NH_4_^+^), 83−99% (NO_3_^−^) under the 5−15 wt% electrode loadings	Low phosphate recovery rate. Negatively charged organics may contribute to fouling and microbial growth	Selective recovery of NH_4_^+^ and NO_3_^−^, PO_4_^3−^	[157]
MCDI	Three-chamber reactor that consists of cathode, anode, and CEM, AEM. Run 1: standard monopolar CEM-DF-120 and AEM-DF-120 (Tianwei Membrane Technology Co., Ltd., Shandong, China) membranes. Run 2: selective to monovalent cations M-CEM (Astom, Japan) and standard monopolar AEM-DF-120 (Tianwei Membrane Technology Co., Ltd., Shandong, China) S = 35.23 cm^2^, Flow rate, W = 5.00 mL min^−1^, U = 1.2 V (charging stage), ttot = 12 h	Synthetic wastewater: with 100 mM NH_4_C1, 50 mM CaCl_2_, and 50 mM MgCl_2_	Product purity of ammonium sulfate increased from around 50% (standard CEM) to 85% (selective CEM). Selective recovery factor for NH_4_^+^ versus another cations: 2. Energy consumption: 2498 J mmol^−1^NH_4_^+^ (standard CEM), 887 mmol^−1^NH_4_^+^ (selective CEM)	Module design and process conditions require optimization	Selective recovery of N^III^	[153]
ECS	ECS (electrochemical stripping) combines electrodialysis and membrane stripping in a three-chamber reactor: cathode//CEM//GPM/anode, where cation exchange membrane, CMI-7000 (Membranes International Inc., Ringwood, NJ) and gas permeable membrane, GPM (CLARCOR, Industrial Air, Overland Park, KS) were used. Catholyte was always 0.1 M NaCl. i = 10 mA cm^2^, U = 2.9 V t = 9 h	(NH_4_)_2_SO_4_ solution imitating municipal wastewater (30 mg (N^III^) L^−1^), leather wastewater (300 mg (N^III^) L^−1^), anaerobic digestate (3000 mg N L^−1^)	Process does not need adding strong base; constant NH_3_ recovery. N^III^ recovery efficiency: 65%; N^III^ removal efficiency: 73%	Back-diffusion of NH_4_^+^, a 2.5-fold decrease in the ammonium flux with an increase in the salinity of the feed solution from 300 to 3000 mg N L^−1^)	Selective recovery of N^III^	[158]
ED	Cathode//CEM//AEM/anode, 1 pair cell with CEM and AEM (Membrane International Inc., Ringwood, NJ, USA). U = 5 V, t = 6 h	Real centrate: 1417 ± 29 mg L^−1^ (TAN), 103 ± 6 mg L^−1^ (PO_4_^3−^), 393 ± 27 mg L^−1^ (Na^+^), 236 ± 21 mg L^−1^ (K^+^), 308 ± 23 mg L^−1^ (Ca^2+^), 1175 ± 48 mg L^−1^ (Cl^−^), 2707 ± 186 mg L^−1^ (TSS), 1663 ± 0.37 mg L^−1^ (COD)	Removal efficiency: 74 ± 4% (N^III^), 60 ± 2% (P^V^). Energy consumption: 17.7 ± 0.6 kWh kg^−1^(N^III^)or 291.3 ± 13.3 kWh kg^−1^ (P^V^)	Loss of almost 30% Cl^−^ due to oxidation at the anode	Recovery of N^III^ and P^V^; reagentless pH shift due to electrode reactions	[159]
ED	Conventional ED stack consisting of 1 pair cell with Fujifilm Type 10 CEM and Fujifilm Type 10 AEM (Fujifilm, Netherlands) or self-produced CEM, AEM membranes. The solution volume in the dilute and concentrate circuits were equal to 1.0 L and 0.3 L, respectively. U = 50 V, t = 360 min	Sewage sludge ash leached by 0.05 M H_2_SO_4_ with PO_4_^3−^ concentration 2.95 g L^−1^	Synthesized membranes demonstrated the same results as commercial one. Recovery factor: 14.75 (PO_4_^3−^) achieved during 30 min	No data available for other components	Recovery and concentration of P^V^	[160]
ED	Conventional ED stack consisting of 4 pair cell with CEM and AEM (Mega, Czech Republic). S = 64 cm^2^ per membrane; W = L h^−1^; U = 6.6 V. The solution volume in the dilute and concentrate circuits equal to 2 and 0.5 L; batch mode; t = 120 h	The real municipal wastewater in the secondary clarifier tank of the CAS system: 67.8 mg L^−1^ (Cl^−^), 100 mg L^−1^ (NO_3_^−^), (113.3 mg L^−1^ (SO_4_^2−^), 68.22 mg L^−1^ (Na^+^), 33.55 mg L^−1^ (K^+^), 52.4 mg L^−1^ (Ca^2+^), 10.19 mg L^−1^ (TOC), 500 mg L^−1^ (TDS), 340 mg L^−1^ (total salinity)	The high water recovery capacity of ED. NO_3_^−^ concentration factor: 4.6 (single-stage); 19.2 (two-stage). Energy consumption: 1.44 kWh kg^−1^ (NO_3_^−^) (single-stage); 4.34 kWh kg^−1^ (NO_3_^−^) (two-stage).	heavy fouling AEMs by organic compounds, compare to CEMs	Recovery and concentration of N^V^	[161]
ED	Conventional ED stack consisting of 5 pair cell with IONSEP-HC-C and IONSEP-HC-A (Iontech, China) membranes. i = 25 mA cm^−2^ (1.25i_lim_^exp^) t = 4 h	A solution with 0.116 g L^−1^ Na_2_HPO_4_·7H_2_O, 0.085 g L^−1^ NaH_2_PO_4_·H_2_O, and 5.2 g L^−1^ Na_2_SO_4_	Electrodialysis in overlimiting current modes provides the separation of sulfates and phosphates. SO_4_^2−^ are transferred through the AEM, while phosphates are converted into phosphoric acid molecules and accumulate in the diluate circuit	AEM degradation: the appearance of macropores between the ion-exchange polymer and the inert binder, loss of mechanical strength, decrease in electrical conductivity and selectivity, etc.	Selective recovery of P^V^	[162,163]
ED	Conventional ED stack consisting of 10 pair cell with PCA SA and PCA SK standard membranes as well as two PCA SC cation exchange end membranes. S = 64 cm^2^. The current density is dynamically adjusted in agreement with the decreasing ion concentration of the diluate, without exceeding the limiting current density	Synthetic solution of the sludge reject water: 6.6 g L^−1^ (NH_4_HCO_3_)	Removal efficiency: 90% (N^III^); Concentration 10 g L^−1^ of NH_4_^+^ is reached. Energy consumption: 5.4M J kg^−1^(N^III^) NH_3_ using as fuel in the solid oxide fuel cell which produces energy13 M J kg^−1^ (N^III^)	Osmosis from the diluate compartment to the concentration compartment and ammonium reverse diffusion take place. About 5% of ammonium accumulating in electrode compartments (using end AEM might prevent it)	Recovery of N^III^ and energy production	[164]
SED	The electrodialysis stack contained five repeating units consisting of 5 PC-MVK membranes, 5 PC-MVA membranes, 5 PC-SA membranes, 4 PC-SK membranes and 2 PC-SC end membranes. From the anode to the cathode, a PC-SK membrane, a PC- MVK membrane, a PC-MVA membrane and a PC-SA membrane were installed in order. All membranes were provided by PolymerchemicAltmeier, GmbH, Heusweiler, Germany. S = 64 cm^2^; U = 7.8 V, W = 10.62 cm s^−1^, Operating time = 140 min	Simulated swine wastewater: 40 mg-P L-1 (NaH_2_PO_4_·H_2_O), 500 mg-N L^−1^ (NH_4_Cl), 100 mg-SO_4_ L^−1^ (Na_2_SO_4_), 400 mg-K L^−1^ (KCl), 60 mg-Mg L^−1^ (MgCl_2_) and 100 mg-Ca L (CaCl_2_)	28.38 kWh/kg PO_4_–P energy consumption (89.6% recovery); energy consumption at 0.783 kWh/kg NH_4_-N (63.2% recovery). Recovered Mg^2+^ and Ca^2+^ during the process can be used for next phosphate precipitation (with dosing 2 mol L^−1^ NaOH)	Current efficiency 30.23% (NH_4_-N), 4.16% (PO_4_–P)	Selective recovery of P^V^ and N^III^	[165]
BMED	Base-BMED stack consisting of 7 pair cells with bipolar (electrically fused AR103 and CR61) and monopolar (CR67) membranes (SUEZ Water Technologies & Solutions, Canada) An AEM (AR 204, SUEZ Water Technologies & Solutions, Canada) was placed next to the cathode while an extra CEM (CR67, SUEZ Water Technologies & Solutions, Canada) was placed to the anode. S = 36,7 cm^2^; U = 30 V, W = 180 mL min^−1^, operating time, t= 60 min	Dewatering centrate: 1188.85 ± 31.5 mg L^−1^ (NH_3_-N); 120.66 ± 3.46 mg L^−1^ (Ca^2+^); 81.66 ± 2.42 mg L^−1^ (Mg^2+^); 101.58 ± 4.24 mg L^−1^(K^+^); 275.21 ± 7.66 mg L^−1^ (Na^+^); pH 7.63 ± 0.08	Ammonia recovery: 60%; removal efficiency: 86,5% (NH_4_^+^); 95.1% (K^+^); 84,0% (Ca^2+^); 63,2% (Mg^2+^); energy consumption: 15.0 kW h kg^−1^N Dewatering centrate as the feed to BMED system did not need an extra pretreatment (e.g., filtration) because AEMs, that are vulnerable to organic fouling, were excluded from the BMED stack design (except for the electrode rinse cell)	5.2% of ammonia was lost during operation; the negligible amount (0.01 g L^−1^) of ammonia was transferred to the electrode rinse solution through AEM located next to the cathode; 82.6–91.8% of Ca^2+^ and 62.6–76.0% of Mg^2+^ (compared with the mass of Ca^2+^ and Mg^2+^ in the feed dewatering centrate) were precipitated on the CEM	Reagentless pH shift for selective recovery of N^III^	[166]
BMED	Tree-compartment-BMED stack consisting of triple cells with bipolar (PCA) and monopolar (PCA SK, PCA Acid-60) membranes (PCCell GmbH, Heusweiler, Germany). S = 62 cm^2^	Synthetic residual streams: sludge reject water or certain industrial condensates: 6.6 g L^−1^ (NH_4_HCO_3_)	TAN removal efficiency: from 85 to 91%; the energy consumption: 19 MJ kg^−1^ (N^III^). Replacing the CEMs by AEMs in the BMED membrane stack decreasing NH_4_^+^ loses	Leakage of hydroxide, diffusion of dissolved ammonia and ionic species from the base compartment to the diluate, which cause the current efficiency decreased from 69 to 54% during batch BMED. 27% of the NH_4_^+^ passes from the diluate solution to the electrode compartment trough CEM	Reagentless pH shift for selective recovery of N^III^	[167]
BMED	Tree-compartment-BMED stack consisting of 1 triple cell with bipolar (BPM-1, BPM-2 self-produced) and monopolar (Fujifilm Type 10, synthesized AEM membranes. The electrode solution: 0.3 M Na_2_SO_4_. i = 10 mA cm^−2^, t = 300 min	Sewage sludge ash leached by 0.05 M H_2_SO_4_: 2.95 g L^−1^ (PO_4_^3−^)	Achieved concentration of phosphoric acid is 0.104 M for BPM-2. (Improving of phosphoric acid production up to 45%). Synthesized membranes demonstrated the same results as commercial	Low phosphoric acid production	Reagentless pH shift for selective recovery of P^V^	[160]
BMED	Tree-compartment-BMED: BPM//AEM//CEM//BPM, base-BMED: BPM//CEM//BPM, acid-BMED: BPM//AEM//BPM. S = 180 cm^2^, I = 3A, Umax < 60 V, t = 330 min	Synthetic wastewater imitating the liquid fraction of animal manure after separation into solid and liquid phases: 4.28 g (NH_4_Cl), 9.90 g L^−1^ ((NH_4_)_2_SO_4_), 2.64 g L^−1^ NaH_2_PO_4_), 5.39 g L^−1^ (CH_3_COONH_4_), 1.33 mL L^−1^ (H_3_PO_4_), 2.64 mL L^−1^ (butyric acid), 2.04 mL L^−1^ (valeric acid)	Consistent application of the base-BMED and the acid-BMED reduced NH_3_ losses. NH_3_ was concentrated up to 16 g L^−1^ in the base solution (close to 99%) but energy consumption was risen to 2.73 MJ against 1.20 MJ for three-compartment- BMED	Tree-compartment-BMED: recovery rate: 44.5% (NH_4_^+^), 81.6% (Cl^−^) 96.0% (PO_4_^3−^); about 18% of NH_3_ passes from the base compartment to the acid one; 70% of energy is consumed by the solution resistance, undesired NH_3_ flux, and concentration polarization phenomena	Reagentless pH shift for N^III^ and P^V^ selective recovery	[168]
BMED + HFMC	Tree-compartment-BMED stack consisting of 4 triple cells with bipolar (BP-IE) and monopolar (CMX, AMX) membranes (Astom, Japan). Each membrane area S =189 cm^2^ HFMC module (Pureseaspring, China). The average flow velocity, V, of the basified wastewater and the acid solution are 2 cm s^−1^ and 1 cm s^−1^), respectively. I= 20 mA cm^−2^	The synthetic wastewater: NH_4_C1 (5000 mg L^−1^), NaCl (2000 mg L^−1^), Na_2_SO_4_ (2000 mg L^−1^) in deionized water	BMED energy consumption: 119.88 kj mol^−1^NH_4_^+^ – N; current efficiency: 80.0%. BMED–HFMC NIII capture ratio: >99%; energy consumption: 111.26 kj mol^−1^ (N^III^) NH_4_^+^ concentration in the wastewater was decreased to <10 mg L^−1^, the achieved concentration of by-product (NH_4_)_2_SO_4_ 139.1 g L^−1^	NH_3_ undergoes leakage from the acid compartment to the salt compartment via AEM owing to coion transport and concentration diffusion; membrane fouling of the complex organic and/or inorganic components in the real wastewater should be overcome	BMED alkalized the wastewater and transform NH_4_^+^ to NH_3_; the MCDI is used to remove ammonia	[169]
BMED+ MCDI	Tree-compartment-BMED stack consisting of triple cell with bipolar (Fumasep FBM, Fuma-Tech Co., Japan) and monopolar (CMX, AMX, Astom, Japan) membranes. S = 17.5 cm^2^. Synthetic seawater (sea salt concentration of 35 g L^−1^.) in the acidic chamber to increase the electrical conductivity. T = 8 h, U = 1.4 V	Synthetic wastewater with 2.5 mM PO_4_^3−^ and 12.5 mM NH_4_^+^	Removing∼89% of phosphorus and∼77% of NH_4_^+^, recovering ∼81% of wastewater. Energy consumption: 3.22 kWh kg^−1^ N. Simultaneously getting struvite and NH_4_^+^ concentrating	Adding MgCl_2_ × 6H_2_O for struvite precipitation	BMED alkalized the wastewater to facilitate struvite precipitation; the MCDI is used to remove NH_4_^+^	[154]

*Conventional electrodialysis (ED)* is characterized by alternating cation and anion-exchange membranes, which form a pair chamber consisting of desalination (DC) and concentration (CC) compartments. This method has been validated for many liquid media including municipal wastewater [161], sewage sludge ash [160], industrial streams [163,170], etc. For example, the use of a two-stage batch regime makes it possible to achieve an almost complete recovery of nitrates and an enhanced nitrate concentration ratio to 19.2 with energy consumption of 4.35 kWh/kg NO_3_^−^ (in terms of nitrates) [161]. It should be noted that the feed solutions are multicomponent and, as a rule, contain several types of anions and cations. Competitive transport of these ions through membranes reduces the efficiency of target components recovery and preconcentration [161].

**Figure 8:**
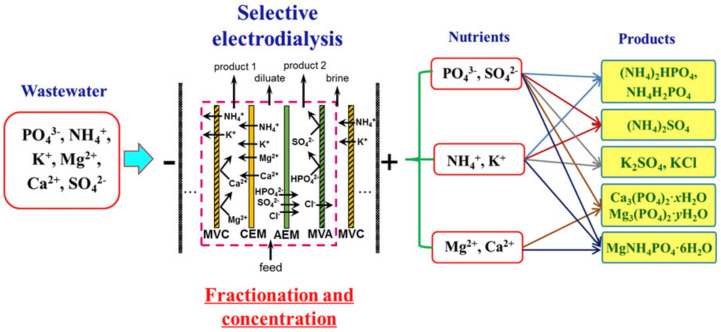
Possible pairwise fractionation and concentration of nutrients from multicomponent solutions using SED. Adapted and modified from [165].

The application of *selectrodialysis (SED)* provides a solution to this problem [171,172,173]. Ye et al. [165] show (Figure 8) that a multicomponent solution can be fractionated into an anionic product stream with multiply charged nutrient anions (PO_4_^3–^ and SO_4_^2–^), cationic product stream with bivalent nutrient cations (Mg^2+^ and Ca^2+^); a monovalent cations (K^+^ and NH_4_^+^) may be concentrated in the brine stream. Moreover, SO_4_^2–^ and Cl^−^ anions are transferred through membranes much easier in comparison to phosphates. In the case of cations, the permeation sequence is: NH_4_^+^ ≈ K^+^ > Ca^2+^ > Mg^2+^ ≈ Na^+^.

**Figure 9:**
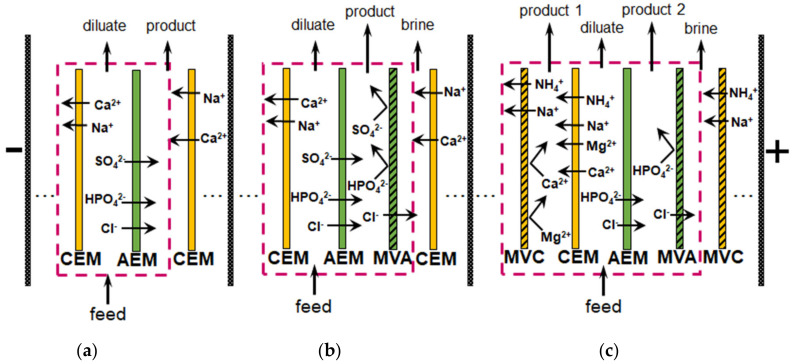
Scheme of repeating units of membrane stacks for conventional ED (**a**), anion selectrodialysis, aSED (**b**), and biselectrodialysis, bSED (**c**). Based on [175].

Unlike conventional ED (Figure 9a), the elementary unit of the membrane stack consists of three (Figure 9b) or four (Figure 9c) compartments. In the case of anion selectrodialysis (aSED), the multicomponent feed solution is fed into a desalination compartment formed by standard CEM and AEM. In an applied electric field, all anions are transferred via the AEM to the target product compartment (Figure 9b). The multiply charged anions remain in this compartment, while the monovalent anions move through the monovalent anion-exchange membrane (MVA) into the concentration compartment. All (mono- and multiply charged) cations are transferred to the same compartment via CEM. The feed solution must have a pH greater than 7 to ensure that the vast majority of phosphates are converted to multiply charged anions, which are rejected by MVA. A cation-exchange membrane (MVC) selective for monocharged cations is introduced into the membrane stack (Figure 9c) to simultaneously obtain a solution enriched in ammonium cations. This process is called biselectrodialysis (bSED). Meesschaert et al. [174] and Ghyselbrecht et al. [175] demonstrated the possibility to selectively recover and concentrate phosphates from a synthetic feed solution as well as from anaerobic sludge blanket reactor effluent (that had previously been nitrified, ultra-filtered, and ultraviolet treated) using first lab-scale and then pilot-scale aSED. The concentrations of phosphates (the product stream), potassium, and nitrates (the brine stream) were 5–6 mmol/L, 150 mmol/L and 90 mmol/L, respectively. A total of 98% of P^V^ was precipitated as calcium phosphate using a lamella separator. The use of the membrane stack configuration as shown in Figure 9b [173] made it possible to provide the initial recovery rate of 0.072 mmol/(m^2^ s) (phosphates) and 1.31 mmol/(m^2^ s) (ammonium). As a result, 70% of phosphates and ammonium have been removed from the digester supernatant.

It should be noted that ED is the only membrane method that makes it possible to simultaneously obtain high-purity P^V^ and recover N^III^ [176] from dilute liquid media, as well as to concentrate these substances to the maximum [170,177]. For example, Wang et al. [178] achieved an 18 fold increase in the concentration of NH_4_^+^ recovering it from the liquid component of pig manure by ED. However, the energy consumption was 202–258 MJ/kg _N_ (in terms of nitrogen). Ward et al. [179] succeeded in concentrating ammonium by a factor of 8.5 with an energy consumption of 18 MJ/kg _N_, comparable to the traditional Anammox process [180]. Recently, Saltworks Technologies Inc.© has commercialized an electrodialysis technology for ammonium concentration from industrial wastewater and landfill liquid effluents to produce cheap fertilizers [181]. These advances in the applied field explain the exponential growth of publications in Scopus on this topic. This growth began in 2005. The total number of publications in Scopus has increased four times (keywords “ammonium OR phosphate AND electrodialysis”) over the past 10 years.

**Figure 10:**
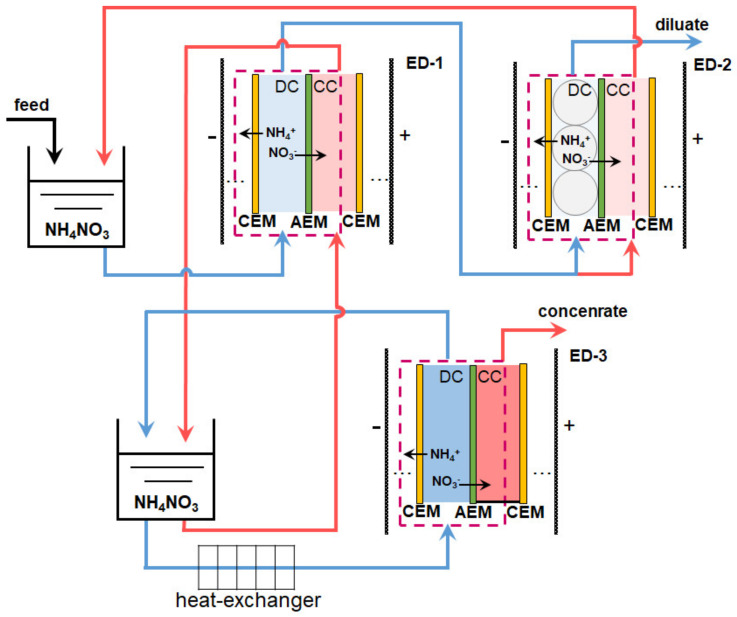
Scheme of ED processing of the condensate of the secondary stream formed during the production of ammonium nitrate. ED-1 is a conventional electrodialyzer, ED-2 is an electrodialyzer-deionizer with a mixture of anion-exchange and cation-exchange resins in desalination compartments, and ED-3 is an electrodialyzer-concentrator with enclosed (non-flow) concentration compartments. Based on [170].

ED is especially good in the final stage of wastewater treatment or in the case of industrial wastewater containing only soluble salts [170,177]. For example, the secondary stream condensate formed during the production of ammonium nitrate contains only NH_4_NO_3_. Melnikov et al. [170] proposed a scheme of three ED modules (Figure 10) for maximum concentration of NH_4_NO_3_ and obtaining pure water. The condensate (feed solution) was pumped from the tank I to DC of conventional electrodialyzer ED-1, the membrane stack of which consisted of alternating CEM and AEM. 90% of the partially desalinated by ED-1 solution entered the flow DC of the electrodialyzer-deionizer ED-2. A monolayer of a mixture of cation- and anion-exchange resins in DC of ED-2 ensured almost complete removal of NH_4_NO_3_ from deionized water. A total of 10% of demineralized by ED-1 water volume was pumped through the ED-2 concentration compartment and then returned to the intermediate tank.

The NH_4_NO_3_ solution from the intermediate tank II of the ED-1 concentration circuit was supplied to the DC of the electrodialyzer-concentrator ED-3, which had enclosed (non-flow) CC. Under the action of an electric field, NH_4_^+^ cations and NO_3_^−^ anions are transferred to the CC. Water is pumped to these compartments only due to osmosis or electroosmosis (as part of the hydration shells of salt ions). The pilot-scale unit, which operated at a mineral fertilizer plant, demonstrated the following characteristics. The demineralized solution contained an order of magnitude less salt and ammonium, and the concentration of NH_4_NO_3_ increased 150 times in the concentrated solution as compared to the feed solution (~1g/L NH_4_NO_3_, 2% NH_3_) at an energy consumption of less than 2.5 kWh/kg. Moreover, the cost of salt separation did not exceed 0.07 Euro/kg, because the membrane stacks consisted of relatively inexpensive heterogeneous MK-40 (LTD Shchekinoazot, Russia), commercial membranes Ralex AMH (MEGA, Czech Republic), or commercial membranes MA-41 (LTD Shchekinoazot, Russia) with lab-made profiled surface [170].

#### 3.7.2. Reagent-Free pH Control for Nutrient Recovery and Conversion

**Figure 11:**
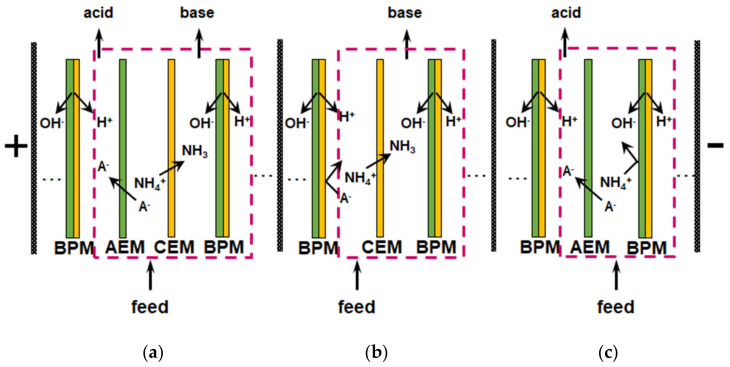
Schematic representation of membrane bipolar electrodialyzers with feed, acid and base (**a**), feed and base (**b**), feed and acid (**c**) repeating units and H^+^/OH^−^ ions generation at the bipolar boundary of the cation and anion-exchange layers of a bipolar membrane. The salt (NH_4_A) contained in the feed solution is converted into acid and alkali as the result of this generation; A^−^ denotes the anions. Based on [168].

Reagent-free acidification and alkalization of solutions is carried out in electrodialyzers containing BPM. The generation of protons and hydroxyl ions takes place at the boundary of the cation- and anion-exchange layers of BPM under the action of applied electric field. An overview of the principles of operation and applications of BPM is given in a recent review [182]. Most often, the elementary unit of membrane stacks consists of two or three compartments (Figure 11). Taking into account the reaction NH_4_^+^ + OH^−^ → NH_3_∙H_2_O, which occurs in an alkaline medium, bipolar membrane electrodialysis (BMED) is very attractive for the reagent-free conversion of ammonium to ammonia [6,166,167,169,183,184].

#### 3.7.3. Integrated Electromembrane Processes

**Figure 12:**
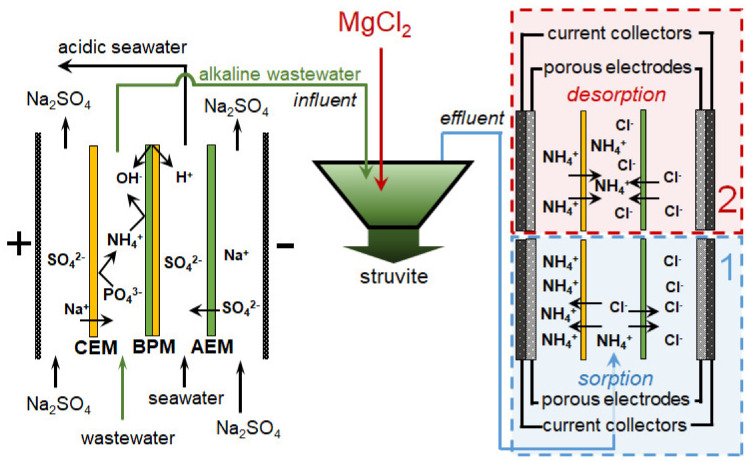
Simultaneous removal and recovery of phosphates and ammonium from wastewater using integrated BMED and MCDI processes. Based on [154].

The combination of BMED with other membrane processes provides a cost-effective, sustainable, and environmentally friendly ammonia recovery and concentration. For example, Gao et al. [154] proposed a hybrid setup that is a combination of BMED and MCDI. The use of this unit (Figure 12) ensured the removal of ~89% P^V^ and ~77% N^III^ from a multicomponent solution (NH_4_Cl and NH_4_H_2_PO_4_) and a decrease in the volume of liquid containing these nutrients by about five times. The energy consumption for this process was 3.22 kWh/kg (N^III^), which is significantly lower compared to the nitrification-denitrification process or the flow-electrode capacitive desalination without IEM.

Xu et al. [185] developed a hybrid system for recover nutrients and energy production from pickled industrial wastewater with concentrated organics, NaCl, ammonia, and P^V^. This system consists of AnD, BMED and SOFC. AnD converted 70% of COD to biogas and methane (~0.051 LCH_4_/g_COD_). BMED enabled liquid phase desalination, acid, and alkaline generation at rates of 0.304, 0.114, and 0.136 mol/h, respectively. Ammonium cations were converted into ammonia without reagents. Fuel cell used recovered biogas and NH_3_/H_2_. The output and the peak power densities were reached, equal to 500 mW/cm^2^ and 530 mW/cm^2^, respectively.

**Figure 13:**
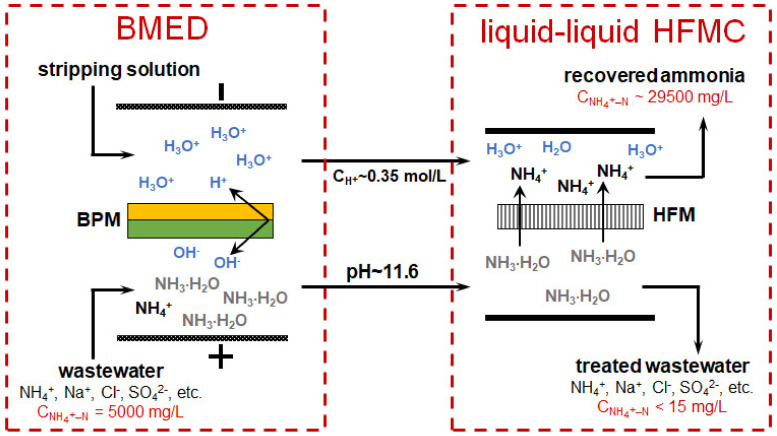
Scheme and details of ion transport in the BMED module for continuous recovery and concentration of N^III^ from wastewater using a combined BMED-liquid-liquid HFMC system. Based on [169].

Yan et al. [169] proposed a combined system for the continuous treatment of wastewater with a high content of mineral salts, including ammonium cations and sulfate anions (Figure 13). The elementary unit of the BMED membrane stack contained three compartments (Figure 11a). Feed solution (wastewater) was circulated through the acid (BPM//CEM) and (base AEM//BPM) compartments. A solution enriched with salt ions contained in the wastewater was circulated through the central salt compartment (CEM//AEM). Basified and acidified wastewater was pumped counter currently through an HFMC from the lumen side and shell side of the membrane, respectively. NH_3_ from the basified wastewater moved via the hydrophobic hollow fiber GSM to the acidified wastewater and accumulated there in the form of (NH_4_)_2_SO_4_, which can be used as fertilizer or for power generation. The ammonium recovery from wastewater has reached 99%. The energy consumption for this combined process was 111.26 kJ/mol_NH4+_, which is much lower than in the case of single HFMC process for ammonia capture. A similar system has also been tested for the processing of urine [186] and municipal solid waste digestate [176].

The combination of conventional ED with BMED enabled to obtain phosphoric acid with preliminary extraction of phosphates by leaching from the sewage sludge ash [160].

The integration of conventional ED and Donnan dialysis (DD) [187] removed up to 89.1% of NH_4_^+^ from simulated high-salinity wastewater. The percentage of ammonium cations recovered was 13.3% and 32.3% higher than that achieved using single modules of conventional ED and DD, respectively. Energy consumptions were reduced by 50.48% as compared to single conventional ED.

In the scientific literature, one can find studies where the process of biochemical transformation of substances from urine into soluble forms of N^III^ and P^V^ is combined with their ED concentration. For example, Monetti et al. [188] proposed a process called “Bio-electroconcentration”. An electroactive microbial community located at the anode is involved in the production of nutrients, which are then concentrated into a liquid fertilizer concentrate using an ED concentration compartment. In this case, the N^III^ recovery efficiency reached of 69.6%—the highest value known to date for bioelectrochemical systems. This device enabled the production of concentrated liquid fertilizers (21.2 ± 0.3 g/L (N^III^), 1.1 g/L (P^V^) and 5.4 ± 0.2 g/L (K^+^)). An average power consumption of 4.1 ± 0.1 kWh/kg _N_ were significantly lower than in the case of the Haber–Bosch process or wastewater treatment using nitrification/denitrification process (~23.5 kWh/kg).

Integration of liquid-liquid HFMC with conventional ED makes it possible to achieve a commercially attractive ammonium concentration (equal to 15–32 wt%) for use in agriculture for fertigation, with an energy consumption of 0.21 ± 0.08 kWh per kg of ammonium salt [126,189].

## 4. Bottlenecks in Nutrient Recovery Processes Using Ion-Exchange Membranes

### 4.1. Low Mass Transfer Characteristics and High Energy Consumption

It should be noted that almost all researchers (Table 1) pay attention to several “bottlenecks” that prevent wider industrial application of IEM processes. First, there are:

(1) lower current efficiency with respect to nitrogen and phosphorus [126,165,174,175,179,181];
(2) lower concentrations of ammonium and phosphate ions in concentrated solutions [175,179,190,191];
(3) higher energy consumption [126,172,192] than those in ED of sodium chloride, potassium nitrate, and other strong electrolyte solutions, which are traditional for electrodialysis processing.

For example, Ghyselbrecht et al. [175] found that the current efficiency with respect to ***phosphates*** in the first 90 min of their transport through standard PS-SA and PC-acid-100-AT monopolar membranes (Polymer-Chemie Altmeier GmbH, Heusweiler, Germany) was 4.3% and 4.8%, respectively, while for other anions of a multicomponent solution the current efficiencies were 18% and 23% (Cl^−^), 45.9% and 45.1% (NO_3_^−^). The selectivity coefficients for these ions (S_Cl-/NO3-_, S_Cl-/SO42-_, S_Cl-/PO43-_) were –0.11, 0.33, 0.40, respectively (a positive value of the coefficient indicates the preferential transfer of chloride anions; a negative value indicates the preferential transfer of another anion). According to [165], the current efficiency and selectivity of membranes with respect to phosphates increase as the total mineralization of the feed solution and its enrichment with phosphoric acid anions decreases. Peculiarities of phosphoric and other polybasic acids anions transport are manifested in an increase in the conductivity [193,194] and diffusion permeability [195,196] of AEMs with dilution of feed solutions; a significant effect of the external solution pH on the sorption of acidic residues of polybasic acids [197]; the appearance of two or more plateaus in the current-voltage characteristics of AEMs [163,198,199]; the complication of the shape of chronopotentiograms as compared to those obtained in strong electrolyte solutions [200,201,202].

Regarding the *ammonium*, van Linden et al. [164] showed that conventional ED has a limitation of the concentration factor and an increase in energy consumption for NH_4_^+^ removal. Shi et al. [168], who systematically investigated the application of BMED to ammonium recovery from animal manure, revealed undesired N^III^ diffusion through BPM from the base to the adjacent compartment. Similar phenomena were observed in a number of other studies—for example, in Ref. [183]. Diffusion through the BPM contaminated the resulting acid with ammonium anions and significantly reduced the recovery of N^III^. In the case of a three-compartment elementary BMED unit (Figure 11a), the recovery rate of NH_4_^+^ was equal to 44.5% against 81.6% (Cl^−^) and 96.0% (PO_4_^3−^) recovery rates. Moreover, the N^III^ flux through the BPM anion-exchange layer was much higher than the possible flux of the NH_4_^+^ cation as a coion.

Many authors pay attention to intensified generation of H^+^ and OH^−^ ions during ED processing of ammonium [203,204,205] and phosphate [198,202,206] containing solutions as compared to that observed in solutions of strong electrolytes. The presence in the feed solution of polybasic organic acid anions, proteins, microorganisms, or their deoxyribonucleic acid [199,207,208,209,210] also enhances WS at the surface of IEM in the DC of ED.

### 4.2. Membrane Fouling and Degradation

FO, MF, UF, gas separation and, especially, IEM in MBR, MMFC and other bioelectrochemical systems, as well as MCDI and ED modules, are subjected to intense chemical and biochemical fouling [90,211,212], which is well known and studied among “bottlenecks” of electromembrane systems and integrated with them processes. Indeed, the nutrient recovery is carried out from liquids that contain a large number of microorganisms or organic and inorganic substances that are a nutritional medium. These substances are adsorbed by IEMs due to electrostatic, ion-dipole, dipole-dipole and other interactions [213,214,215]. In addition, concentration polarization and local changes in pH can lead to scaling of inorganic substances. For example, Guo et al. [216] found precipitation of amorphous calcium carbonate and struvite on membranes after ED treatment of wastewater.

**Figure 14:**
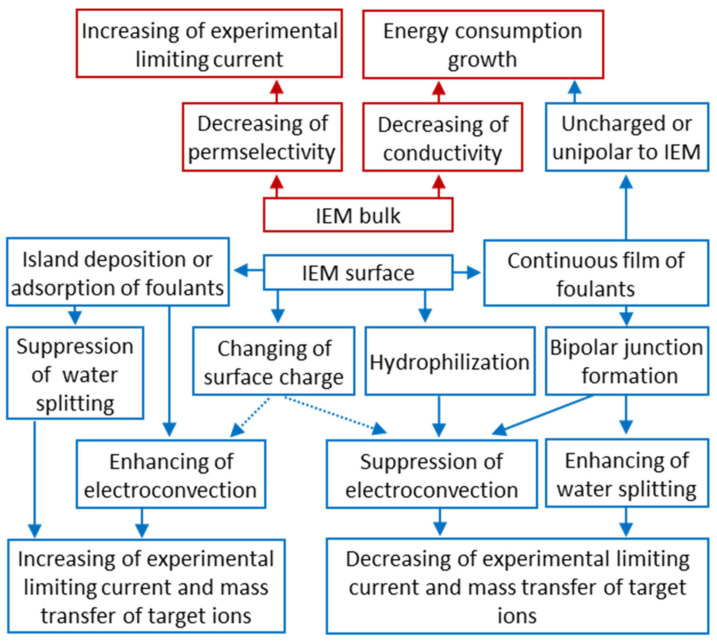
Schematic representation of the changes in the bulk and surface of IEMs caused by fouling and the effect of these changes on the most important characteristics of membrane processes in the applied electric field and in its absence. Reproduced from [214].

A detailed analysis of various fouling, scaling, biofouling mechanisms and the impact of these phenomena on the characteristics of IEM bulk and surface, as well as on their transport characteristics (conductivity, diffusion permeability, permselectivity) and on the phenomena accompanying concentration polarization (WS, electroconvection (EC), etc.) is made in recent reviews [64,80,213,214,217] and summarized in Figure 14. Therefore, we will not analyze these phenomena in detail.

**Figure 15:**
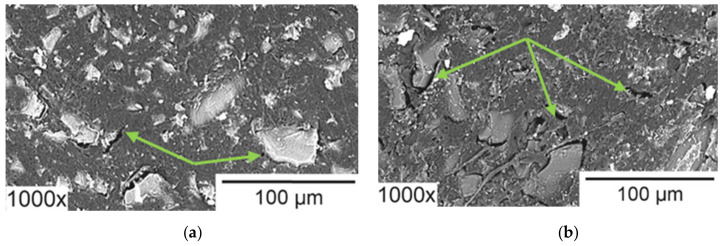
SEM images of heterogeneous ion-exchange membrane IONSEP-HC-A (Iontech, China) before (**a**) and after (**b**) its operation in ED desalination of solution containing 0.116 g/L Na_2_HPO_4_∙7H_2_O, 0.085 g/L NaH_2_PO_4_∙7H_2_O and 5.2 g/L Na_2_SO_4_. Arrows point to cavities between the ion-exchange and inert materials. Reproduced with permission from [162]. Copyright 2022 Elsevier.

**Figure 16:**
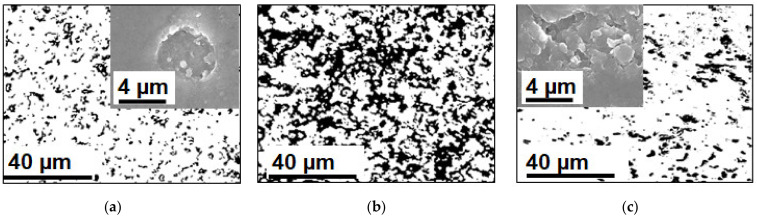
Contrasted optical images of swollen AMX-Sb membranes after 300 h of operation in ED desalination of 0.02 M NaCl (**a**), NaH_2_PO_4_ (**b**) and NH_4_Cl (**c**) solutions. Black color corresponds to an inert binder PVC on the membrane surface. SEM images of the surface of dry membranes after 180 h of operation are presented in the insets.

Note that a number of researchers pay their attention to the rather rapid degradation of heterogeneous [162] and homogeneous [218] AEM in the ED processing of solutions containing phosphoric acid or ammonium anions. In both cases, the operation of membranes in overlimiting current modes leads to the transformation of some fixed groups from quaternary amines into weakly basic secondary and tertiary amines, as well as to the destruction of the polymer matrix. This electrochemical degradation of polymers results in the appearance on the surface of heterogeneous membranes of cavities between the ion-exchange and inert materials (Figure 15). Additionally, a network of shallow slit-like cavities that are filled with flakes of exfoliated ion-exchange material (case of NH_4_Cl) or deeper cavities (case of NaH_2_PO_4_) with polyvinyl chloride (PVC) (the inert filler) on their walls (Figure 16), may be formed. As a result, the AEM conducting surface fraction decreases; the membrane conductivity and selectivity decrease; the WS at AEM surface increases; the EC in the solution adjacent to the AEM reduces [162,218]. These changes lead to a shortening of the life cycle of anion-exchange membranes in nutrient recovery processes as compared to processes carried out in strong electrolyte solutions, such as NaCl [219].

## 5. Fundamentals of Phosphates and Ammonia Transport in Electromembrane Systems

The reasons for the bottlenecks in nutrient recovery processes (see Section 4) have been the subject of scientific debate since the first attempts to use ion-exchange membranes in MBR, MMFC, BES, DD, MCDI, ED, BMED for N^III^, P^V^ recovery, and concentration. By analogy with strong electrolytes, researchers attribute the problems to osmotic or electroosmotic dilution of solutions in the concentration circuits of membrane stacks, back-diffusion intensification caused by the salt concentration gradient between the diluate and the concentrate stream, and also to steric hindrance caused by large sizes of phosphate ions [126,157,164,172,179,181,190,191,192,220]. In recent years, it has been realized that NH_4_^+^ − NH_3_ and phosphoric acid species represent a special class of substances that enter the protonation–deprotonation reactions with water and, therefore, their structure and electric charge depend on the pH of the medium. This property is actively used in various membrane processes for nutrients recovery. However, it is also the reason for the differences in their transport as compared to strong electrolytes (NaCl) in systems with IEM.

### 5.1. Phosphate Containing Solutions

Zhang et al. [221] found that the selectivity of AEMs to phosphoric acid anions depends not only on the size (hydraulic radius) of the transported anions, but also on the pH of the feed solution. The authors of Ref [175] pay attention to the fact that the process of batch ED of a multicomponent phosphate-containing solution is accompanied by acidification of this solution in the desalination circuit and alkalization in the phosphate concentration circuit. Moreover, the less the pH changes in these compartments as compared to the initial value, the higher the current efficiency for P^V^. For the studied standard AEMs, current efficiency increased in the series: PS-CA < Fujifilm Type I < PC-acid-100 OT (Polymer-Chemie Altmeier GmbH, Heusweiler, Germany). Based on these data, Giselbrecht et al. [175] hypothesized the following factors explaining the behavior of the studied membrane systems.

(1) The radii of large and strongly hydrated phosphoric acid anions exceed the radii of other anions; therefore, phosphates have more steric hindrances during their transport in AEMs.
(2) A multicomponent nutrient solution with pH 6.2–7.5 contains H_2_PO_4_^−^ anions, which are deprotonated in standard AEMs and transferred as doubly charged anions. The initial solution with pH 8.0 and higher is enriched in doubly charged HPO_4_^2–^ anions, which move through the AEM without deprotonation. Therefore, the current efficiency increases, and the pH of the solutions in the desalination and concentration compartments does not undergo significant changes, in contrast to more acidic feed solutions.

**Figure 17:**
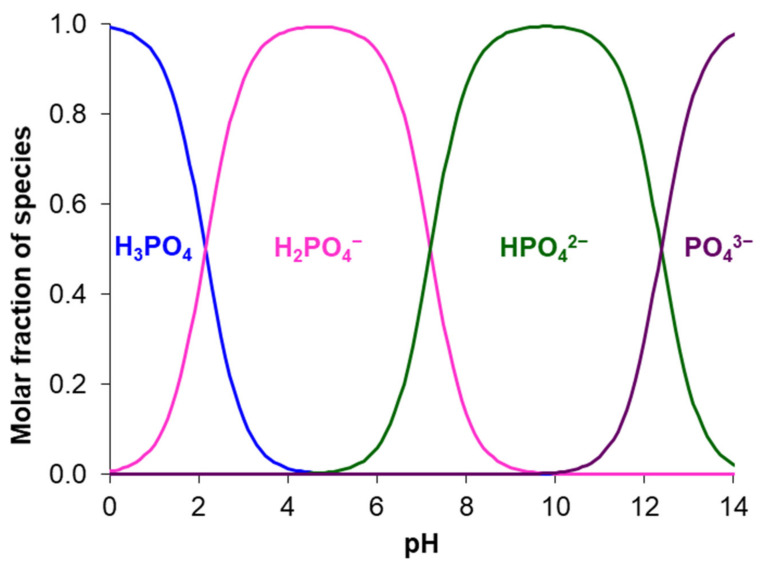
Distribution of mole fractions of phosphoric acid species depending on aqueous solution pH. Reproduced from [207].

A group led by Nikonenko is developing a similar concept. Using the experimental method of color indication [207,215] and mathematical modeling [193,222,223] it has been shown that even in the absence of an electric field, the pH of the AEM internal solution is 3–4 units higher in comparison to the external one. The reason for this difference is the Donnan exclusion of coions [224], including protons, which are the product of the protonation-deprotonation reactions internal solution of AEM. These reactions may involve water, weakly basic fixed groups, phosphoric acid species, and other ampholytes if they are present in the feed solution. Thus, the charge of phosphoric acid species depends on pH due to protonation-deprotonation reactions (Figure 17).

**Figure 18:**
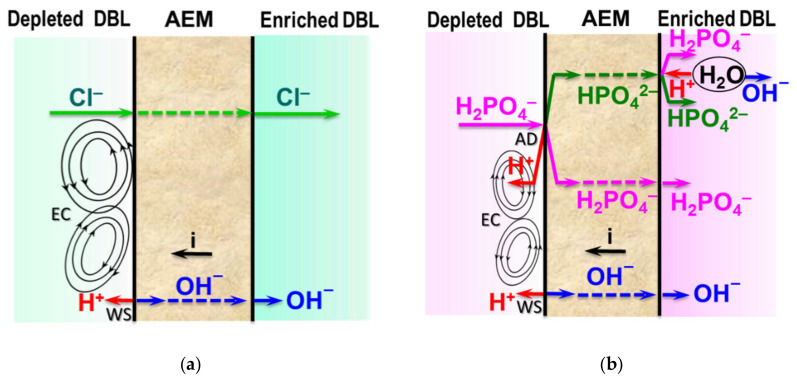
Scheme of proton and hydroxyl ions generation in the system AEM/NaCl (**a**) and AEM/NaH_2_PO_4_ (**b**) solution. WS: water splitting; AD: the acid dissociation mechanisms, EC: electroconvection.

Therefore, entering the AEM, a part of the H_x_PO_4_^(3−x)−^ anions loses a proton (if any) and increases the electric charge, as is schematically demonstrated for the H_2_PO_4_^−^ anion in Figure 18. The proton is excluded into the depleted solution adjacent to the AEM. The doubly charged anion HPO_4_^2−^ moves towards the opposite membrane boundary, crosses it, and ends up in a solution whose pH is lower than in AEM. The result of the HPO_4_^2−^ anion protonation reactions with the participation of water is the generation of singly charged H_2_PO_4_^−^ anions and hydroxyl ions. This space-separated generation of H^+^ and OH^−^ ions is called the “acid dissociation mechanism” (AD) [207]. The AD mechanism is typical for salts of polybasic acids and takes place at any current density. In contrast, the well-known mechanism [225] of the generation of H^+^ and OH^−^ ions with the participation of fixed groups of the membrane WS is realized in solutions of any electrolytes only in overlimiting current modes.

The multiply charged anions transport through the AEM (instead of singly charged H_2_PO_4_^−^) leads to an increase in the current density. At the same time, the total partial flux of P^V^, which is the target component in ED, depends insignificantly upon these transformations. The rate of transfer of singly charged anions from the bulk solution to the AEM/depleted diffusion boundary layer (DBL) interface controls the flux of P^V^ (the same as in the case of strong electrolytes, for example, NaCl). The limiting current, *i_lim_^Lev^* (which characterizes the achievement of the minimum concentration of any type of counterions at the AEM/depleted DBL interface), can be theoretically estimated using the modified Leveque equation [226]:

(1) $$i_{\lim}^{Lev}=\frac{F}{\delta^{Lev}}\sum_{k=1}^{2}\left(1-\frac{z_{k}}{z_{A}}\right)D_{k}z_{k}c_{k}^{0},$$

(2) $$\delta^{Lev}=0.68\;h{\left(\frac{LD_{ter}}{h^{2}V_{0}}\right)}^{1/3},$$

(3) $$D_{ter}=\left[\left(1+\left|\frac{z_{1}}{z_{A}}\right|\right)D_{1}N_{1}+\left(1+\left|\frac{z_{2}}{z_{A}}\right|\right)D_{2}N_{2}\right]\cdot t_{A},$$

where *D_k_*, *z_k_* and *c_k_^0^* are the diffusion coefficient, charge, and molar concentration of counterion *k*, respectively (*k* = 1, 2); *z_A_* is the charge number of the coion common for both counterions, *D_ter_* is the diffusion coefficient of tertiary electrolyte, which consists of two counterions and one coion, *δ^Lev^* is average DBL calculated within the framework of the convective-diffusion model for an empty chamber [227], $N_{i}=\frac{z_{i}c_{i}^{0}}{z_{A}c_{A}^{0}}$ is the equivalent fraction of counterion *i* in the bulk solution. The *c_k_^0^* concentrations are calculated using the equations expressing the equilibriums between different species of a polybasic acid salt with known AD constants, *K_i_*, if the pH of the solution and the cation concentration are given. Gally et al. [202] and Chandra et al. [197] use similar approaches to determine the limiting currents in the case of AEM in multicomponent feed solutions. Note that *i*_lim_*^Lev^* gives an idea of the upper limit of the currents that provide maximum current efficiency due to diffusion, migration, and convective counterions transport.

**Figure 19:**
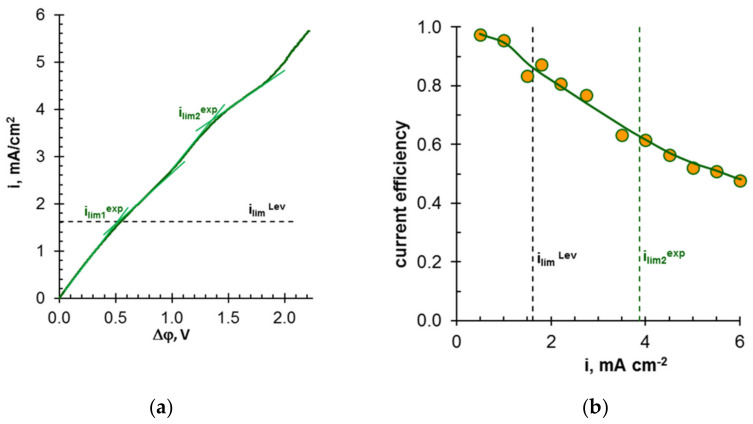
Current-voltage curves (**a**) and dependence of P^V^ current efficiency upon current density (**b**), obtained in the Fujifilm Type X/0.02 M NaH_2_PO_4_ solution system (Fujifilm, Netherlands) (pH = 4.6). The dashed lines show the values of the limiting currents calculated by Equations (1)–(3) and found from the experimental current-voltage curve as shown in fragment (**a**). Based on [198].

**Figure 20:**
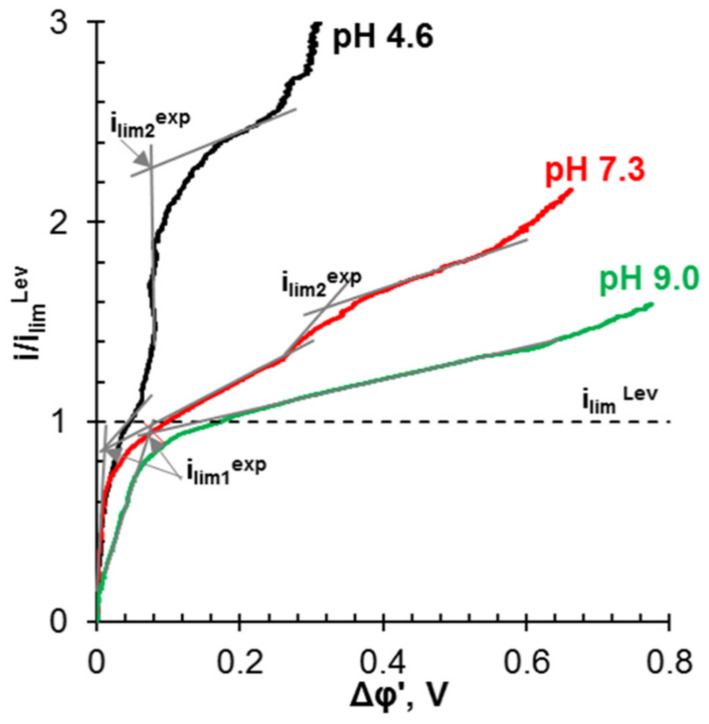
Current-voltage curves of an AX membrane (Astom, Yamaguchi, Japan) in 0.02 M Na_x_H_(3−x)_PO_4_ solutions with pH 4.6, 7.3, and 9.0. The currents are normalized to *i_lim_^Lev^*, calculated for each solution using Equations (1)–(3). The ohmic component is subtracted from the total potential drop. Reproduced with permission from [207]. Copyright 2022 Elsevier.

A consequence of the increase in the charge of the H_2_PO_4_^−^ anion in the membrane is the fact that *i*_lim_*^Lev^* turns out to be two or more times lower than i_lim2_^exp^ [207,228] (Figure 19), which can be determined from a well-visualized plateau in the current-voltage characteristics (Figure 19a and Figure 20). Two different “limiting” current densities are possible in the case of ampholyte-containing solutions. The first one, i_lim1_^exp^, is related to critical decreasing of electrolyte concentration at the membrane surface and reaching maximum electrolyte diffusion flux. This current is identical to that which occurs in membrane systems with a strong electrolyte (e.g., NaCl) and can be estimated using modified Leveque Equation (1). The second limiting current corresponds to state where an AEM is saturated with doubly charged anions in the case of phosphate containing solution. It is found that only an elusive plateau appears at CVC of a membrane system at i = i_lim1_^exp^. Resistance of the system increases noticeably at i = i_lim2_^exp^ and a well-detected horizontal plateau appears in the CVC. Thus, determining of i_lim1_^exp^ from experimental current-voltage characteristics is often difficult for ampholyte-containing systems, and i_lim2_^exp^ is mistakenly using for calculation of optimal current mode for electrodialysis. Moreover, the transport numbers of H^+^ ions in a depleted NaH_2_PO_4_ solution near the AEM surface turn out to be significantly higher compared to the case of NaCl [198]. For example, at a current density of 1.5 *i*_lim_*^Lev^*, the proton transport numbers are 0.38 (NaH_2_PO_4_) and 0.11 (NaCl) for the AMX/0.02 M feed solution system [207]. An increase in pH leads to an increase in the proportion of doubly and/or triply charged phosphoric acid anions in the feed solution. As a result, the difference between the composition of electric charge carriers in solution and AEM decreases. Accordingly, the difference between the limiting current i_lim2_^exp^ (Figure 20) recorded from the experimental current-voltage curve and the theoretical limiting current *i*_lim_*^Lev^* found from Equation (1) decreases.

Enrichment of the solution at the AEM/depleted DBL interface with protons due to the implementation simultaneously of two mechanisms of their generation (AD and WS) causes a decrease in the space charge density and leads to a reducing of EC as compared to strong electrolytes [201,229]. As a result, the increase in the mass transfer of phosphoric acid anions is less significant than in the case of NaCl solution in intense current modes.

The development of the AD mechanism and EC in the case of phosphate-containing systems depends not only on the feed solution pH, but also on the ion-exchange capacity and membrane thickness [207], its conductive surface fraction [228], the rate constant of protonation-deprotonation reactions [230], and other factors, which require further study.

Note that the phenomena described above (the scheme is shown in Figure 18) can be used to separate phosphates and anions that do not participate in the protonation-deprotonation reactions. Indeed, intense protons generation caused by AD and WS mechanisms in highly overlimiting current modes leads to conversion of the phosphates to a non-charged phosphoric acid in ED desalination compartments while the SO_4_^2–^ anions are transferred through the AEMs to the concentration compartments [162].

The Donnan exclusion of protons as coions and the enrichment of AEM with multiply charged anions of phosphoric acid (and other polybasic acids) also occur in the absence of an electric field. For example, estimates based on mathematical modeling [207] show that the gel phase of an AMX membrane (Astom, Yamaguchi, Japan) equilibrated with a 0.02 M Na_x_H_(3−x)_PO_4_ solution (pH 4.6) contains 38.2% of doubly charged HPO_4_^2–^ anions, while in solution the concentration of these ions is 0.2%. Doubly charged HPO_4_^2–^ anions are more hydrated, have a large Stokes radius as compared to H_2_PO_4_^−^ anions and can interact simultaneously with two AEM fixed groups [193]. Therefore, the transport of HPO_4_^2–^ anions in the membrane is accompanied with stronger steric hindrances than the transport of H_2_PO_4_^−^ and, moreover, the more mobile Cl^−^ ions. As a result, the AEM conductivity in phosphate-containing solutions decreases compared to the conductivity in solutions of strong electrolytes (NaCl). An increase in the external solution pH leads to an increase in the portion of multiply charged anions in membranes, reducing their conductivity in moderately dilute and concentrated solutions (c > 0.1 M). At the same time, this conductivity increases in dilute solutions (c < 0.1 M) [193] due to the enhancement of the Donnan exclusion of protons [224]. Apparently, the growth factor of membrane conductivity with increasing counterion charge (æ~$z_{1}^{2}$ [224]) prevails over the factor of its decrease caused by steric hindrance of counterion transport. Similar phenomena take place in the presence of polybasic organic acid species in feed solution [194]. Multicharged counterions attract more coions to the gel phase compared to singly charged counterions. Therefore, the AEM diffusion permeability increases with dilution of the solution simultaneously with the increase in the membrane conductivity [196].

**Figure 21:**
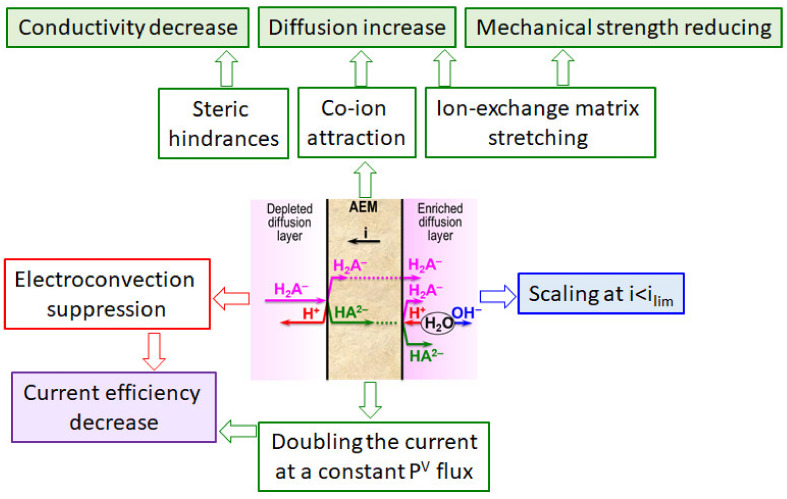
Factors determining the mass transfer characteristics of AEM in solutions containing phosphoric acid species.

The scheme presented in Figure 21 summarizes all the known factors that affect the transport of H_x_PO_4_^(3−x)−^ anions in systems with AEMs.

### 5.2. Ammonium Containing Solutions

**Figure 22:**
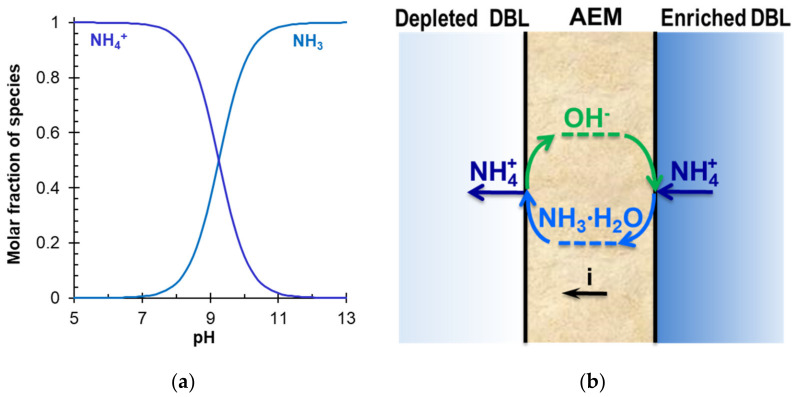
Distribution of mole fractions of NH_4_^+^ and NH_3_ species in aqueous solutions depending on pH (**a**) and a schematic representation of the mechanism of ammonium cations “facilitated” diffusion through an anion-exchange membrane due to higher pH values in AEM than into the external solution (**b**). Reproduced with permission [205]. Copyright 2022 Elsevier.

Using analogy with strong electrolytes, many researchers explain the relatively low current efficiency [126,181] and high energy consumption [126,192], as well as the problems with achieving high concentrations of ammonium cations in ED concentration compartments by the insufficient selectivity of CEMs, for which NH_4_^+^ is counterion [179,190,191]. Indeed, an increase in selectivity and a decrease in the diffusion permeability of CEM lead to a decrease in the ammonium cations fluxes [231] and a decrease in energy consumption for the ED recovery of ammonium salts [232]. At the same time, the so-called “facilitated” diffusion of ammonium cations through AEM [169,205,233] (Figure 22b) can also significantly affect the mass transfer characteristics of the membrane systems with ammonium-containing solutions.

Indeed [233], the measured integral coefficient of AEM diffusion permeability in the system H_2_O/AMX/1 M NH_4_Cl solution are 1.7 times higher than the value obtained under the same conditions (pH 5.4 ± 0.2 at 25 °C) in the system H_2_O/AMX/1 M KCl solution. Such an increase in diffusion permeability cannot be caused by the transport of the NH_4_^+^ cation as a coion. Melnikova et al. proposed a mathematical model [233], which takes into account protonation-deprotonation reactions of water and the NH_4_^+^ cations in the AEM and adjacent DBLs. Based on the calculations using this model, the following mechanism for increasing the ammonium coions transport (Figure 22b) can be proposed. The ammonium cations enter the AEM from the side of the CC in ED. A part of the NH_4_^+^ is deprotonated and converted to NH_3_ molecules due to the high pH value inside the membrane. The NH_3_ molecules diffuse through the AEM towards the side facing the more dilute solution. Crossing this boundary, NH_3_ molecules enter a more acidic environment, are protonated, and are again converted Into NH_4_^+^ cations. In this case, OH^−^ ions are released and returned to the AEM surface adjacent to the concentrated solution. As already mentioned, the more alkaline medium in the membrane in comparison to the external solution is due to the Donnan exclusion from membrane of protons (as coions [224]), which are formed as a result of WS, protonation–deprotonation of fixed groups, etc. Direct measurements using with color indicators [205] and calculations [233] show that the pH of the internal AEM solution is 4–5 units higher than the pH of the external ammonium-containing solution.

The data of chronopotentiometry, electrochemical impedance spectroscopy and voltammetry, solutions pH measurements and determining counterion transport numbers in AEM, [204,205] conform ammonia participation in H^+^ and OH^−^ ions generation at the AEM/depleted DBL interface. In overlimiting current modes, the alkalinity of the AEM internal solution and the solution at the AEM/CC interface increases even more significantly due to the inclusion of WS. Thus, the operation of AEM in overlimiting current modes should increase the diffusion of NH_3_, whose molecules have zero charge and small size. The similar situation is observed in the case of BMED, when NH_3_ diffuses through the cation- and anion-exchange layers of BPM from the base compartment into the acid or DC [168,183].

### 5.3. Membrane Degradation

**Figure 23:**
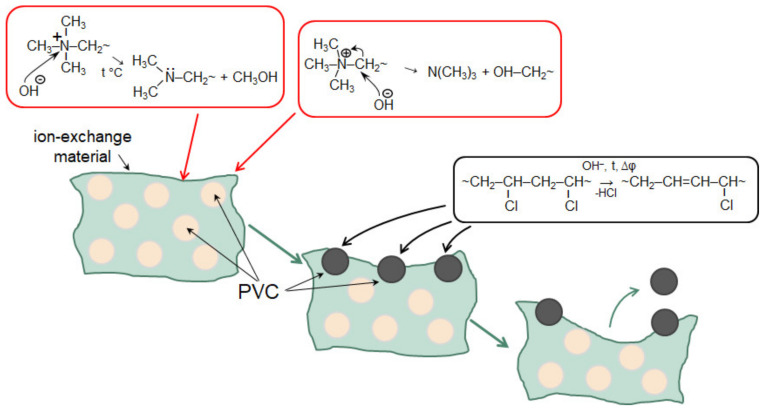
Schematic representation of the degradation of anion-exchange membranes produced by the paste method.

As already mentioned in Section 4.2, the causes of membrane fouling are considered in sufficient detail in many original papers and reviews, for example, in [64,80,213,214]. Therefore, we will briefly dwell only on the more intense degradation of IEMs during their operation in solutions containing phosphoric acid and/or ammonium anions. It was already mentioned in Section 5.1 and Section 5.2 that the AEM internal solution is enriched with hydroxyl ions as compared to the external solution in underlimiting current modes. In addition, the presence of ammonium cations and phosphoric acid anions in the feed solutions stimulates H^+^, OH^−^ ions generation, which results in an even greater local pH misbalance at the interface AEM/depleted DBL. Moreover, the operation of membranes in intensive current modes produces a high electric field strength at the AEM/depleted DBL interface. The combination of these factors stimulates the occurrence of several chemical reactions (Figure 23) involving the ion-exchange material. First, this is the nucleophilic attack of quaternary amines by hydroxyl ions [234], which are always present in aqueous solutions due to the water dissociation. In addition, it is a sequence of reactions called Stevens rearrangement [235] and other reactions summarized in the review [236]. The result of these reactions is the transformation of some quaternary amino groups into secondary and tertiary amines, the elimination of fixed groups from the polymer matrix, and the breaking of the carbon chains of the polymer matrix, which is a copolymer of polystyrene and divinylbenzene. The degradation of PVC, which is an inert filler and reinforcing cloth in membranes made by the paste method [237], for example, AMX, AMX-Sb (Astom, Japan), is also attacked by hydroxyl anions in an electric field. The 2E elimination reaction mechanism [238] results in the conversion of PVC into polyenes [239,240], which are black (Figure 16). Apparently, the latter circumstance led to the renewal of the assortment of membranes produced by Astom, Yamaguchi, Japan [240]. Polyethylene, which is often an inert binder in heterogeneous membranes, also undergoes degradation at high electric fields and at local changes in pH in intense current modes [241,242]. More intense generation of H^+^ and OH^−^ ions in solutions containing ammonium ions or phosphoric acid anions, apparently, contributes to enhance membrane degradation as compared to solutions of strong electrolytes.

**Figure 24:**
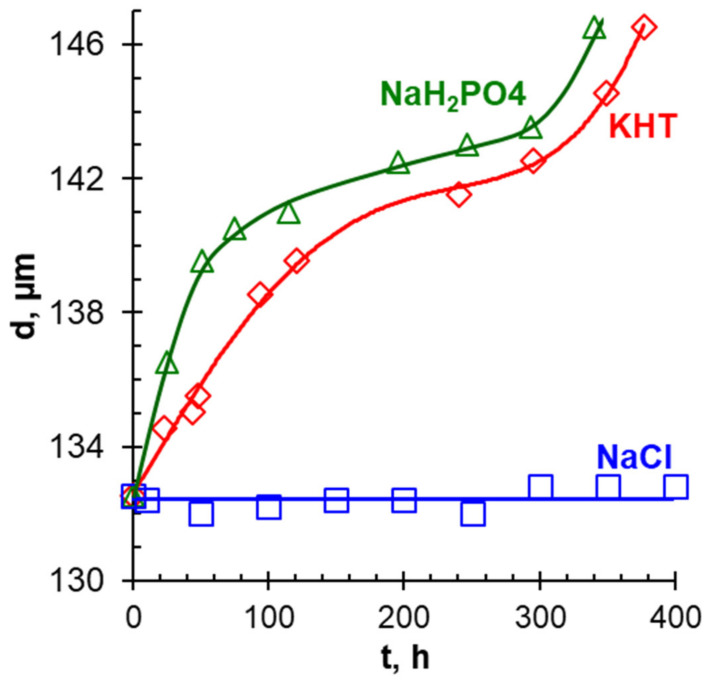
The AMX-Sb anion-exchange membrane (Astom, Yamaguchi, Japan) thickness versus soaking time in 0.02 M sodium chloride (NaCl), potassium hydrotartrate (KHT), and sodium hydrogen phosphate (NaH_2_PO_4_) solutions. Reproduced from [196].

Note that the phosphoric acid anions, as well as the anions of other polybasic acids (citric, tartaric, carbonic, etc.) often found in nutrient contained liquids, are highly hydrated [243]. Getting into the AEM pores, these substances cause an increase in the osmotic pressure on the pore walls in comparison with that observed, for example, in NaCl solutions [224]. As a result, the membrane ion-exchange matrix is stretched; the effective pore radius (and the water content) as well as the membrane thickness increase (Figure 24).

This phenomenon occurs both in the case of homogeneous (AMX-Sb) and heterogeneous (MA-41, Shchekinoazot, Russia; FTAM-EDE, FUMATECH BWT GmbH, Heusweiler, Germany) membranes [196]. Drying such AEMs before performing SEM imaging of their surface results in the appearance of gaps between the ion-exchange materials and the inert binder, as shown in Figure 15.

## 6. Innovations in Nutrient Recovery Processes with Ion-Exchange Membranes

### 6.1. Enhancement Nutrient Mass Transfer

*Phosphates.* To reduce steric hindrance in phosphate transport, Zhang et al. [221] proposed to use thin porous AEMs with minimal selectivity to monocharged anions.

Recent knowledge (summarized in [175,207,229]) on the mechanisms of phosphate transport in IEM systems (see Section 5.1) suggests that an increase in the pH of the feed phosphate-containing solutions may improve the P^V^ current efficiency. However, in this case, steric hindrance will increase, since only doubly and/or triply charged phosphoric acid anions will be transported through the AEM. In addition, an increase in pH can adversely affect the AEM exchange capacity due to the deprotonation of weakly basic groups. Therefore, this hypothesis requires careful testing.

The use of IEMs coated with layers that selectively sorb phosphates is another promising direction for increasing the current efficiency in the recovery of phosphates. For example, Petrov et al. [244] propose to use CEM modified with high surface area adsorbent with iron oxide nanoparticles (Fe_3_O_4_NPs) coated with polyhexamethylene guanidine. This polyelectrolyte enters into a selective interaction with phosphate [245]. In addition, intermediate layers of polyethyleneimine and poly (styrene sulfonate) are deposited to increase the surface roughness and the charge density of the modifying layer. This layer faces the cathode. At the first stage of the electroadsorption process, phosphates are sorbed by this layer. At the second stage of the process, the direction of the electric field is reversed, and desorption of phosphates takes place.

NH_4_^+^ − NH_3_. The use of CEM selective to monocharged cations can significantly increase the current efficiency with respect to NH_4_^+^ cations recovered from multicomponent solutions that contain multiply charged cations along with NH_4_^+^. For example, Wang et al. [153] used a MVC manufactured by Astom, Japan. This made it possible to increase the purity of the product obtained by the MFCDI method to 85% as compared to 50% achieved using standard CEM. At the same time, the portion of NH_4_^+^ cations among other co-existing cations doubled. Promising results are also obtained by applying the ion-selective polyelectrolyte layer to the electrodes. Thus, the use of guanidinium-functionalized polyelectrolyte-coated carbon nanotube (Gu-PAH/CNT) electrode [153] provided selective adsorption of phosphate ions and the repulsion of coions due to the strong electrostatic interactions of the NH protons of Gu groups with phosphate ions as well as hydrogen-bond formation.

Improvement of BMED was mainly aimed at selecting the most efficient designs of membrane stacks. Shi et al. [168] showed that the use of base-BMED (Figure 11b) resulted in a decrease in the base compartment pH as compared to tree-compartment-BMED (Figure 11a) due to the H^+^ ions transport through the CEM from the dilute compartment to the base compartment. As a result, NH_4_^+^ recovery rate increases due to reduced diffusion of NH_3_ through the BPM. A decrease in the NH_4_^+^ concentration in the feed solution after the base-BMED stage causes some decrease in the diffusion of NH_3_ through the BPM in the case of acid-BMED (Figure 11c). Therefore, the authors of [168] proposed the sequential use of base-BMED and acid-BMED modules instead of a single tree-compartment-BMED. This technical solution increased the time of the electrodialysis process but provided almost 99% recovery of NH_4_^+^ from the feed solution versus 40% in the case of tree-compartment-BMED. Taking into account the mechanism of N^III^ transport through AEM (Figure 22), it can be concluded that the positive effect of the two-stage BMED caused by the absence of monopolar AEMs in the membrane stacks at the first (base-BMED) stage of electrodialysis. Meanwhile, Shi et al. [168] emphasized that the loss of N^III^ caused by the diffusion of NH_3_ molecules through the BPM is still the biggest challenge of nutrient recovery in BMED.

Recall that the flux of OH^−^ ions, which goes towards NH_4_^+^ coions in the BPM anion-exchange layer, promotes the formation of NH_3_, which easily diffuses into the compartments adjacent to the BPM [246]. The same can be said about the monopolar AEM, which generates H^+^ and OH^−^ ions at the AEM/depleted DBL interface in overlimiting current regimes. In the case where the conventional batch ED is performed at a given current density, the i/i_lim_ ratio increases as nutrients are recovered from the feed solution. The effect of OH^−^ ions on the AEM enrichment with NH_3_ molecules and/or multiply charged phosphoric acid anions increases (NH_4_^+^ + OH^−^ → NH_3_; H_x_PO_4_^(3−x)−^ + OH^−^ → H_(x−1)_PO_4_^(3−(x−1)−^). Van Linden et al. [164] proposed to carry out the batch ED process at a given i/i_lim_ ratio; that is, to reduce the current density in proportion to the degree of the NH_4_HCO_3_ solution desalination. The use of such a dynamic current density led to a decrease in the osmotic flow into the concentration compartment from 10% to 2% (with a difference in NH_4_^+^ concentrations in CC and DC equal to 7 g/L) and an increase in the concentration factor from 4.5 (the fixed current density) to 6.7 (dynamic current density).

Note that the end membranes of membrane stack that bordering the electrode compartments can also affect product purity and current efficiency. For example, van Linden et al. [167] have shown that up to 27% of NH_4_^+^ is transferred from the dilute compartments of tree-compartment-BMED to the cathode compartment when it is bounded by a monopolar CEM. The replacement of cation-exchange end membranes with anion-exchange end membranes markedly reduced these losses. Meanwhile, Guo et al. [166] showed that a small amount of ammonium ends up in the cathode compartment even when an anion-exchange end membrane is used. Thus, solving the problem of ammonium transport through monopolar AEMs or BPM anion-exchange layers is still an acute problem and requires additional research efforts.

### 6.2. Prevention of Fouling and Membrane Degradation

Fouling and biofouling are counteracted in several ways. The first one is the preliminary separation of the liquid phase and dispersed particles, including viruses and bacteria, using MF or UF membranes, as well as the preliminary treatment of processed media with ultrasound and/or ultraviolet radiation [90].

**Figure 25:**
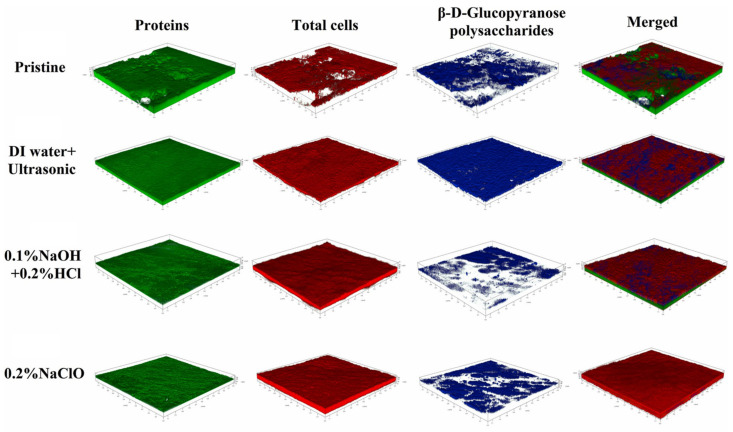
Confocal laser scanning microscopy image of fouled (pristine) FO membrane and the same membrane after ultra-sonication and chemical cleaning using 0.1% NaOH/0.2% HCl or 0.2% NaClO. The images show distribution of individual foulants (green for proteins; red for total cells; blue for polysaccharides) and their superposition (merged). Reproduced from [90].

The second one is a periodic cleaning of membranes using acids, alkalis, perchlorates, and other oxidizing agents [90] or enzymes [9,114,247,248,249]. Xue et al. [90] clearly demonstrated the effectiveness of such treatment (Figure 25). They showed that the layer of foulants (proteins, microorganism cells, polysaccharides) on the surface of the pristine FO membrane used in wastewater treatment reached 80 µm. Ultrasonic are mechanically shaking off microorganisms thanks to the ultrasonic vibrations. The use of 0.1% NaOH and 0.2% HCl, as well as 0.2% NaClO reduced the thickness of the foulant layer to 30 µm. Moreover, acid and alkali primarily acted on organic substances covering microorganisms, while the stronger oxidizing agent NaClO destroyed both organic substances and microorganisms.

Thirdly, active research is underway to impart anti-fouling characteristics to ion-exchange and other membranes [250,251,252]. The increasing of the AEMs surface hydrophilicity [250], as well as changing the surface charge to the opposite of the charge of fixed groups [251,252] are the most common ways. In addition, the synthesis of new membranes with anti-organic fouling properties [87,88] are promising for solving this problem.

**Figure 26:**
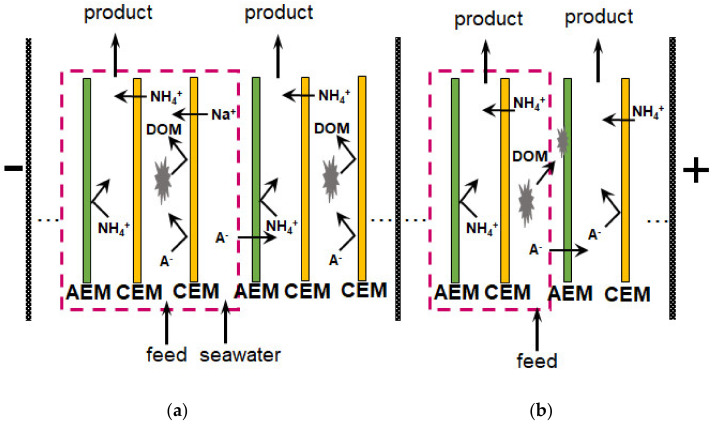
ED with additional CEM, which prevent the fouling of AEM by dissolved organic matter (DOM) (**a**) and conventional ED (**b**). Based on [255].

Fourthly, reverse ED [253] and pulsed electric fields [254] are used. In addition, membrane stacks are being improved in a way to reduce the content of anion-exchange materials in them. These materials contain amino groups, which are the food for microorganisms. For example, Meng et al. [255] proposed a membrane stack for NH_4_^+^ recovery from digested sludge centrate. The desalination compartment of this stack contains additional CEM (Figure 26a).

Isolation of AEM from negatively charged bacteria and anions of organic substances (amino acids, acidic residues of carboxylic acids, etc.), which enter the electrostatic interactions with membrane fixed groups, made it possible to reduce fouling, increase current efficiency and reduce energy consumption by 14% as compared to those achieved in case of conventional ED (Figure 26b). Similar success was achieved by Guo et al. [166], who used only CEMs in BMED.

## 7. Conclusions

The transition to the circular economy, where waste becomes a source and, in particular, nutrients are produced from waste, is an indispensable condition for maintaining the sustainable development of humankind. Low-reagent and resource-saving membrane technologies enable such a transition due to: (i) feasibility of effective and energy-saving recovery, fractionation, and concentration of nutrients, in particular P^V^ (phosphoric acid species) and N^III^ (NH_4_^+^ − NH_3_) compounds, (ii) opportunities to organize a continuous and conjugated chain of transformation of phosphorus and nitrogen compounds into nutrient forms convenient for processing.

Ion-exchange membranes are gradually becoming an essential element of many integrated (hybrid) membrane systems. Such systems include membrane biochemical reactors and membrane biochemical fuel cells; electrochemical systems for low-reagent precipitation of phosphorus-containing fertilizers, as well as for electro-oxidation of organic impurities or nitrogen-containing compounds conversion; installations for Donnan and neutralization dialysis, capacitive deionization, various types of electrodialyzers for feed solution demineralization, high concentration of nutrients, reagent-free production of stripping solutions, conversion of ammonium ions to ammonia, as well as nutrient salts to acids and alkalis, and other.

Unfortunately, many of these promising membrane processes are still under development at the laboratory stage. A few bottlenecks prevent more intensive implementation of these processes in the industry. These are fouling of ion-exchange membranes, contamination of products with impurities, lower current efficiency, higher energy consumption, and lower nutrient concentrations in the brine compared to those achieved by processing solutions containing only strong electrolytes (e.g., NaCl).

In recent years, there has been a qualitative leap in understanding the transport mechanisms of phosphates and NH_4_^+^ – NH_3_ species in systems with ion-exchange membranes. In particular, it has been found that bottlenecks are often caused by the deprotonation of nutrient species in anion-exchange membranes, in which the internal solution pH is always higher than the pH in the feed solution. In the case of phosphates, such deprotonation causes the participation in the transport through the AEM of anions having a higher (negative) electrical charge than in the feed solution. In the case of ammonium-containing solutions, this phenomenon promotes a significant back diffusion of ammonia through monopolar AEMs (and not through CEMs, as previously thought), as well as through the anion-exchange layers of BPMs. In both cases, the participation of phosphates and ammonium in protonation-deprotonation reactions causes an increase in the generation of H^+^ and OH^−^ ions by anion-exchange membranes and a reduction of electroconvection in solution near the surface of these membranes.

New knowledge opens up prospects for further improvement of nutrients recovery using ion-exchange membranes. Suppression of the transformation of singly charged phosphoric acid anions into doubly (or triply) charged, as well as a decrease in the diffusion of ammonia through the anion-exchange membrane, can further intensify useful mass transfer for more successful extraction of nutrients. Replacement of AEM with CEM in membrane stacks, if possible; optimization of electric current regimes and feed solution pH; and new approaches to the modification of ion-exchange membranes are already yielding encouraging results. We hope that this review will be useful and will make a certain contribution to accelerating the transition of industrial production to a new economy through the improvement of waste processing, where ion-exchange membranes will be actively involved.

## Nomenclature

AD	Acid dissociation
AEM	Anion-exchange membrane
AnD	Anaerobic biochemical digestion
AnMBR	Anaerobic membrane bioreactor
aSED	Anion selectrodialysis
BMED	Bipolar membrane electrodialysis
BPM	Bipolar membrane
bSED	Biselectrodialysis
CC	Concentration compartment
CEM	Cation-exchange membrane
COD	Chemical oxygen demand
DBL	Diffusion boundary layer
DC	Desalination compartment
DD	Donnan dialysis
DOM	Dissolved organic matter
EC	Electroconvection
ED	Electrodialysis
FC	Freeze concentration
FO	Forward osmosis
GSM	Gas separation membrane
HFM	Hollow fiber membrane
HFMC	Hollow fiber membrane contactor
IEM	Ion-exchange membrane
LLMC	Liquid-liquid membrane contactor
MBR	Membrane bioreactor
MCDI	Membrane capacitive deionization
MD	Membrane distillation
MF	Microfiltration
MFCDI	Membrane capacitive deionization with flow electrodes
MFC	Microbiological fuel cell
MMFC	Membrane fuel cell
MVA	Anion-exchange membrane selective for monocharged anions
MVC	Cation-exchange membrane selective for monocharged cations
NF	Nanofiltration
OMFC	Osmotic microbiological cell
PP	Polypropylene
PTFE	Polytetrafluoroethylene
PVC	Polyvinyl chloride
PVDF	Polyvinylidene fluoride
RO	Reverse osmosis
SED	Selectrodialysis
SOFC	Solid oxide fuel cell
TAN	Total ammonia nitrogen
TKN	Total Kjeldahl nitrogen
TSS	Total suspended solids
UF	Ultrafiltration
VMS	Vacuum membrane stripping
WS	Water splitting
WWTP	Wastewater treatment plant
